# Disentangling horizontal and vertical Pleiotropy in genetic correlation estimation: introducing the HVP model

**DOI:** 10.1007/s00439-025-02762-w

**Published:** 2025-09-16

**Authors:** Lamessa Dube Amente, Natalie T. Mills, Thuc Duy Le, Elina Hyppönen, S. Hong Lee

**Affiliations:** 1 Australian Centre for Precision Health, University of South Australia, Adelaide, SA 5000 Australia; 2 UniSA Allied Health and Human Performance, University of South Australia, Adelaide, SA 5000 Australia; 3 South Australian Health and Medical Research Institute, Adelaide, SA 5000 Australia; 4 Epidemiology department, Jimma University, Jimma, 378 Ethiopia; 5 Discipline of Psychiatry, University of Adelaide, Adelaide, South Australia 5000 Australia; 6 UniSA STEM, University of South Australia, Mawson Lakes, SA 5095 Australia; 7 UniSA Clinical and Health Sciences, University of South Australia, Adelaide, SA 5000 Australia

## Abstract

Genome-wide genetic correlation studies have demonstrated widespread shared genetic architecture between complex traits, yet the impact of vertical pleiotropy on these genetic correlation estimates remains unclear. To address this, we propose the Horizontal and Vertical Pleiotropy (HVP) model, designed to disentangle horizontal from vertical pleiotropy effects. This approach provides unbiased genetic correlation estimates specifically attributed to horizontal pleiotropy. Through simulations, we verify that the HVP model corrects biases introduced by vertical pleiotropy—particularly the causal influence of exposure on outcomes—across various scenarios, improving the accuracy of heritability and genetic correlation estimates. Vertical pleiotropy biases genetic variances and covariances, influencing essential estimates such as SNP-based heritability and genetic correlation in traditional methods. By addressing these biases, the HVP model enhances accuracy in parameter estimation. Real data analysis shows that horizontal pleiotropy significantly contributes to genetic correlations between metabolic syndrome (MetS) and traits such as type 2 diabetes, C-reactive protein (CRP), sleep apnoea, and cholelithiasis, whereas vertical pleiotropy is more relevant between body mass index (BMI) and MetS, and MetS and cardiovascular diseases. These findings suggest that action on modifiable factors like lowering BMI may effectively reduce MetS risk, while CRP—though not causative—serves as a useful marker in risk prediction through horizontal pleiotropic genes. These results confirm the HVP model’s relevance and utility in revealing the complex genetic architecture underlying traits such as metabolic syndrome, highlighting its potential to inform precision healthcare.

**Supplementary Information:**

The online version contains supplementary material available at 10.1007/s00439-025-02762-w.

## Introduction

Genome-wide genetic correlation studies quantify the shared genetic architecture between complex traits, a concept known as horizontal pleiotropy (Ni et al. 2018; Rheenen et al. 2019; Zhang et al. 2021). These methods rely on individual-level data and genome-wide association study (GWAS) summary statistics to estimate genetic correlations and reveal significant relationships between traits (Ni et al. 2018; Bulik-Sullivan et al. 2015). Methods based on Restricted maximum likelihood (REML) (Lee et al. 2012a, b), including Genome-wide Complex Trait Analysis (GCTA) (Yang et al. 2011, 2013), MTG2 (Lee and van der Werf 2016), and BOLT-REML (Loh et al. 2015), are commonly employed for this purpose, especially in traits with polygenic architectures.

Previous studies have reported substantial genetic correlations across various diseases. For instance, studies published in Science (2018) and Nature Genetics (2013 & 2015) demonstrated substantial genetic correlations among psychiatric disorders, emphasizing the widespread impact of shared genetic effects (Bulik-Sullivan et al. 2015; Cross-Disorder Group of the Psychiatric Genomics et al. 2013; Brainstorm et al. 2018). Similarly, significant genetic correlations between metabolic syndrome (MetS) and multiple complex traits have been reported (van Walree et al. 2022). However, current approaches do not distinguish whether these correlations are driven by horizontal or vertical pleiotropy, limiting our understanding of the underlying mechanisms.

Pleiotropy generally manifests in two primary forms: vertical and horizontal (Ni et al. 2018; van Rheenen et al. 2019; Zhang et al. 2021; Hemani et al. 2018). Vertical pleiotropy occurs when a genetic variant influences one trait, which in turn causally affects another trait (van Rheenen et al. 2019; Lee et al. 2012a, b; Hackinger and Zeggini 2017; Sivakumaran et al. 2011). In contrast, horizontal pleiotropy occurs when genetic factors independently affect two traits, reflecting shared biological processes (Solovieff et al. 2013; Chesmore et al. 2018). Disentangling these two forms is essential for clarifying genotype-to-phenotype relationships and disease mechanisms.

This distinction has important practical implications. For example, vertical pleiotropic effects may not drive changes in the target trait through approaches like functional targeting, gene knockdown, or CRISPR-based gene editing unless the intermediary trait is also involved. In contrast, a modifiable exposure will not affect the outcome in the absence of vertical pleiotropy. Without recognizing this, practical applications such as targeted interventions may be hindered.

Current genetic correlation methods such as REML or Linkage Disequilibrium Score Regression (LDSC) (Bulik-Sullivan et al. 2015; Lee et al. 2012a, b; Yang et al. 2011; Cross-Disorder Group of the Psychiatric Genomics et al. 2013; Brainstorm et al. 2018; van Walree et al. 2022) implicitly assume that observed correlations reflect shared genetic effects alone. However, vertical pleiotropy can also induce genetic correlations that reflect phenotypic causality rather than shared genetic etiology. This conflation presents a critical limitation, as it obscures biological mechanisms and may undermine the predictive utility of polygenic models, especially when causal pathways or mediator traits differ across populations.

To address these issues, we propose the Horizontal and Vertical Pleiotropy (HVP) model, a novel approach designed to disentangle horizontal and vertical pleiotropy while accounting for causal relationships between traits. The HVP method aims to provide unbiased estimates of genetic correlation attributed to shared genetic effects, thereby enhancing both causal inference and the biological interpretability. We evaluate the performance of the HVP model through extensive simulations and apply it to estimate the genetic correlation between MetS and related traits using UK Biobank data. The analysis reveals that horizontal pleiotropy drives genetic correlations between MetS and traits such as type 2 diabetes, C-reactive protein (CRP), sleep apnea, and cholelithiasis, while vertical pleiotropy links body mass index (BMI) with MetS and MetS with cardiovascular diseases. These findings suggest that lowering BMI could effectively reduce MetS risk, while CRP serves as a useful risk biomarker. This highlights the HVP model’s utility in uncovering the complex genetic architecture of MetS and its potential implications for healthcare research.

## Method

### Ethical statement

This study utilized data from the UK Biobank (http://www.ukbiobank.ac.uk/*)*, which follows a rigorously reviewed scientific protocol approved by the Northwest Multi-centre Research Ethics Committee, the National Information Governance Board for Health & Social Care, and the Community Health Index Advisory Group. Participants provided electronic consent, completed questionnaires on socio-demographic and lifestyle factors, and underwent physical measurements. Blood samples (approximately 45 ml) were collected and stored for various analyses, including genetic, proteomic, and metabolomic studies. Access to UK Biobank data was granted under project number 14575.

### Statistical model: horizontal and vertical Pleiotropy

**Fig. 1:**
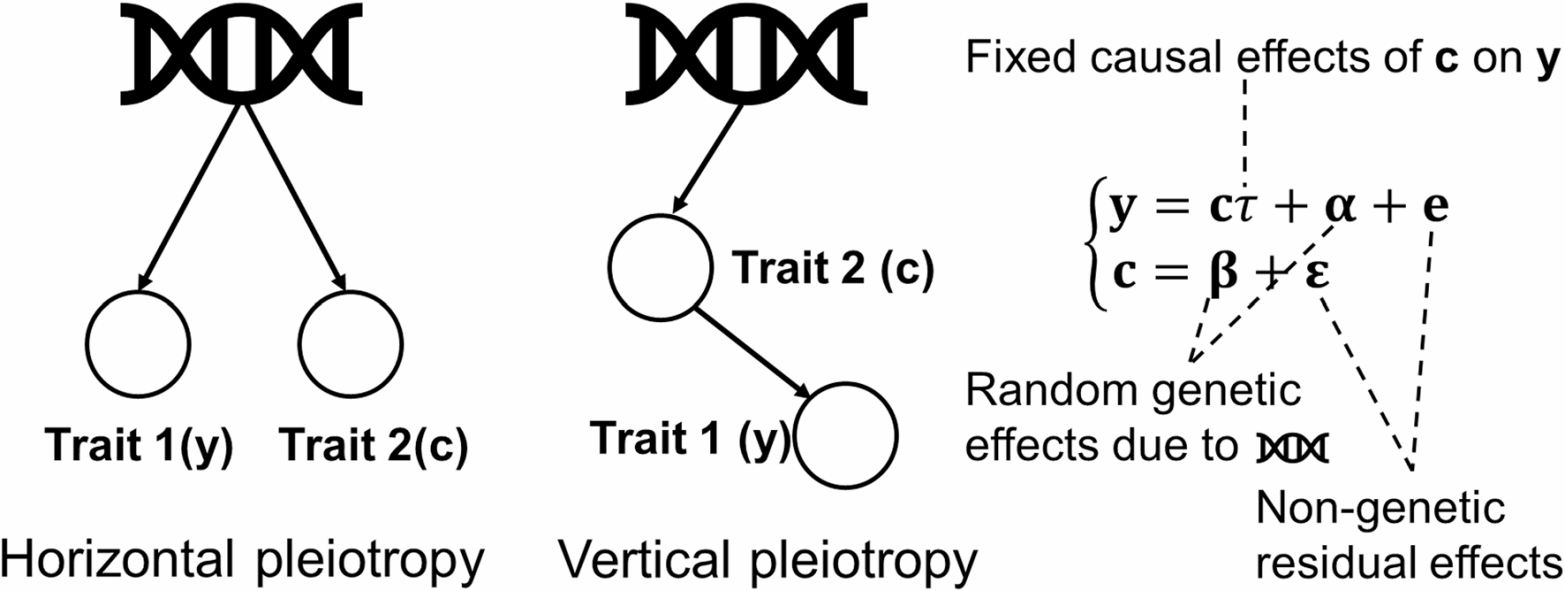
Illustration of the HVP model: Relationship between genotypes and trait 1 (y) and trait 2 (c) under horizontal and vertical pleiotropy. In horizontal pleiotropy, genotypes directly affect the phenotypes of both traits. In vertical pleiotropy, genotypes are associated with trait 2 sharing the same causal pathway to trait 1, i.e., there is a causal relationship between **c** and **y**

Simultaneous consideration of horizontal and vertical pleiotropy (HVP) is feasible within a bivariate linear mixed model (see Fig. 1). In this HVP model, the genetic effects influencing trait 1 are shared with those influencing trait 2, and, concurrently, the phenotypes of trait 1 are partially determined by the phenotypes of trait 2 (Eq. 1).

1 $$\left\{\begin{array}{c}\mathbf{y}=\mathbf{c}\tau\:+\varvec{\upalpha\:}+\mathbf{e}\\\:\mathbf{c}=\varvec{\upbeta\:}+ \varvec{\epsilon} \:\:\:\:\:\:\:\:\:\:\end{array}\right.$$

where **y** and **c** represent traits of interest (outcome vs. exposure), *τ* represents fixed causal effects of trait 2 (**c**) on trait 1 (**y**), and **α** and **β** denote random genetic effects associated with traits **y** and **c**, respectively. Additionally, **e** and **ϵ** encompass non-genetic residual effects pertaining to traits **y** and **c**, respectively. The genetic effects of both traits traverse two distinct paths, namely horizontal and vertical pleiotropy.

Assuming that **c** is standardised with a mean of zero and a variance of one, the estimated *τ* can be expressed as:

2 $$\widehat{\tau\:}=cov\left(\mathbf{y},\mathbf{c}\right)=cov\left(\varvec{\upalpha\:},\:\varvec{\upbeta\:}\right)+cov\left(\mathbf{e}, \varvec{\epsilon} \right)+\tau\:\cdot\:\left(var\right(\varvec{\upbeta\:})+var( \varvec{\epsilon} \left)\right).$$

This equation highlights the potential bias in estimating *τ* due to genetic and residual covariances. Additionally, the variances of **y** and **c** can be written as:

3 $$\begin{aligned} var\left(\mathbf{y}\right)= &var\left(\varvec{\upalpha}\right)+var\left(\mathbf{e}\right)+{\tau}^{2}\cdot\left(var\left(\varvec{\upbeta\:}\right) +var\left( \varvec{\epsilon} \right)\right) \\ &+2\cdot\:\tau\:\cdot\:\left(cov\left(\varvec{\upalpha\:},\varvec{\upbeta\:}\right)+cov\left(\mathbf{e}, \varvec{\epsilon} \right)\right) \end{aligned}$$

4 $$var\left(\mathbf{c}\right)=var\left(\varvec{\upbeta\:}\right)+var\left( \varvec{\epsilon} \right)$$

It is important to note that a mis-specified model without accounting for vertical pleiotropy is often used in genetic analyses. This model can be expressed as

5 $$\left\{\begin{array}{c}\mathbf{y}=\mathbf{g}+\varvec{\upgamma\:}\\\:\mathbf{c}=\varvec{\upbeta\:}+ \varvec{\epsilon} \end{array}\right.$$

where $\mathbf{g}=\varvec{\upalpha\:}+{\uptau\:}\cdot\:\varvec{\upbeta\:}$ and $\varvec{\upgamma\:}=\mathbf{e}+{\uptau\:}\cdot\: \varvec{\epsilon}.$ Subsequently, the following equations can be derived:

6 $$var\left(\mathbf{g}\right)=var\left(\varvec{\upalpha\:}\right)+{\tau\:}^{2}\cdot\:var\left(\varvec{\upbeta\:}\right)+2\cdot\:\tau\:\cdot\:cov\left(\varvec{\upalpha\:},\varvec{\upbeta\:}\right)$$

7 $$cov\left(\mathbf{g},\varvec{\upbeta\:}\right)=cov\left(\varvec{\upalpha\:},\varvec{\upbeta\:}\right)+\tau\:\cdot\:var\left(\varvec{\upbeta\:}\right).$$

This clearly illustrates the biased estimates of genetic variances within a univariate context, comparing *var*(**g**) with *var*(**α**). The deviation is a consequence of both $\tau\:$ and *cov*(**α**,**β**). Importantly, this bias extends to genetic covariances, influencing key parameters, including heritability and genetic correlation (refer to Results section).

Leveraging established Mendelian randomization (MR) methods, we anticipate the accurate classification of genetic variants pertaining to horizontal and vertical pleiotropy (Hackinger and Zeggini 2017; Slob and Burgess 2020; Davey Smith and Hemani 2014). This classification enables an unbiased estimate of *τ*. Subsequently, the remaining two key parameters (*cov*(**α**,**β**), *cov*(**e**,**ϵ**)) can be accurately inferred by solving multiple equations that elucidate relationships between the two traits in terms of genetic and residual components. The procedural breakdown is as follows:

#### Estimation of causal effect

##### GREML approach

Starting with Eq. (1), genetic components are decomposed into two distinct subsets: one specific to horizontal pleiotropy (**α**_1_, **β**_1_) and the other to vertical pleiotropy (**α**_2_, **β**_2_) as illustrated in Fig. 1. Specifically, we define:

$$\varvec{\upalpha}={\varvec{\upalpha}}_{1}+{\varvec{\upalpha}}_{2}\:\text{and}\:\varvec{\upbeta}={\varvec{\upbeta}}_{1}+{\varvec{\upbeta}}_{2}.$$

The genetic component for trait 1, under vertical pleiotropy, is expressed as:

$${\mathbf{g}}_{2}={\varvec{\upalpha\:}}_{2}+\tau\:\cdot\:{\varvec{\upbeta\:}}_{2}$$

Thus, the genetic covariance under vertical pleiotropy is:

$$cov({\mathbf{g}}_{2},{\varvec{\upbeta\:}}_{2})=cov({\varvec{\upalpha\:}}_{2},{\varvec{\upbeta\:}}_{2})+\tau\:\cdot\:var\left({\varvec{\upbeta\:}}_{2}\right)$$

Since ​ **α**_2_ and **β**_2_ are independent by construction, cov(**α**_2_, **β**_2_) = 0, and therefore:

$$\tau\:=cov({\mathbf{g}}_{2},{\varvec{\upbeta\:}}_{2})\:/\:var\left({\varvec{\upbeta\:}}_{2}\right)$$

The values of *cov*(**g**_2_, **β**_2_), and *var*(**β**_2_) can be obtained using the conventional GREML (Lee and Werf 2016) method (i.e. Equation (5) if it is based on the SNPs classified as vertical pleiotropy variants. As depicted in the subsequent equations, the $\tau\:$ value is used to differentiate variance components attributed to horizontal and vertical pleiotropy.

##### MR approach

In practice, robust MR methods can be used to estimate an unbiased value of $\tau\:$ (Slob and Burgess 2020, Zhu et al. 2018). To guide researchers in selecting appropriate methods, we recommend following established best practices in MR analysis ((Hemani et al. 2018; Burgess et al. 2019), with a particular focus on approaches that are robust to horizontal pleiotropy. Among these, MRLOVA (Amente et al. 2025) is designed to accommodate invalid instruments through an EM-based framework. We also applied methods from different model classes, including Contamination Mixture (Burgess et al. 2020) and MRMix (Qi and Chatterjee 2019) both of which use mixture modelling to handle heterogeneous instrument validity. These approaches offer complementary robustness features and are well suited to sensitivity analysis. We encourage the use of multiple robust MR methods in combination with sensitivity analyses to evaluate the stability of causal inference under different assumptions.

#### Correction of genetic covariance

From Eq. (7), the genetic covariance attributed horizontal pleiotropy is:

8 $$cov\left(\varvec{\upalpha\:},\varvec{\upbeta\:}\right)=cov\left(\mathbf{g},\varvec{\upbeta\:}\right)-\tau\:\cdot\:var\left(\varvec{\upbeta\:}\right).$$

here, var(**β**) and *cov*(**g**,**β**) are estimated using the conventional bivariate GREML method (Eq. 5) based on genome-wide SNPs.

#### Estimation of standard error for the corrected genetic covariance

The standard error (se) of the corrected genetic covariance, $cov(\varvec{\upalpha\:},\varvec{\upbeta\:})$, can be derived using the delta method. Based on Eq. (8), the corrected genetic covariance is a function of $cov\left(\mathbf{g},\varvec{\upbeta\:}\right)$ and $var\left(\varvec{\upbeta\:}\right)$, assuming that $\tau\:$ is estimated without error, i.e. *f*(*cov*(**g**,**β**), *var*(**β**))= $cov\left(\mathbf{g},\varvec{\upbeta\:}\right)-\tau\:\cdot\:var\left(\varvec{\upbeta\:}\right)$. Then, the delta method approximates the standard error of the corrected genetic covariance as:

9 $$se\left(f\right)=\:\sqrt{\varvec{\theta\:}^{{\prime\:}\:}\varvec{\Omega\:}\varvec\theta\:\:}.$$

where $\varvec{\theta\:}^{{\prime\:}\:}=(\frac{\partial\:\text{f}}{cov(\varvec{g},\varvec\beta\:)}$, $\frac{\partial\:\text{f}}{var\left(\varvec\beta\:\right)}$) is the derivative of *f* with respect to the variance components and $$\varvec{\Omega }=\left[\begin{array}{ll}var\left(cov\left(\mathbf{g},\varvec{\upbeta }\right)\right), & cov(cov\left(\mathbf{g},\varvec{\upbeta }\right),\text{v}\text{a}\text{r}\left(\varvec{\upbeta }\right)) \\ cov(cov\left(\mathbf{g},\varvec{\upbeta }\right),\text{v}\text{a}\text{r}\left(\varvec{\upbeta }\right))& var\left(\text{v}\text{a}\text{r}\left(\varvec{\upbeta }\right)\right)\end{array}\right]$$ is the covariance matrix of the variance components. Each element of **Ω** can be obtained from the information matrix generated by the conventional bivariate GREML method (Lee and van der Werf 2016).

#### Correction of genetic variance

Similarly, the biased estimate of genetic variance in Eq. (6) can be corrected as:

$var\left(\varvec{\upalpha\:}\right)=var\left(\mathbf{g}\right)-{\tau\:}^{2}\cdot\:var\left(\varvec{\upbeta\:}\right)-2\cdot\:\tau\:\cdot\:cov\left(\varvec{\upalpha\:},\varvec{\upbeta\:}\right)$ which is equivalent to

Substituting from Eq. (8):

10 $$\begin{aligned} var\left(\varvec{\upalpha\:}\right)= & var\left(\mathbf{g}\right)-{\tau\:}^{2}\cdot\:var\left(\varvec{\upbeta\:}\right)-2\cdot\:\tau \\ & \cdot\:\left[cov\right(\mathbf{g},\varvec{\upbeta\:})-\:\tau\:\:\cdot\:var(\varvec{\upbeta\:}\left)\right].\end{aligned}$$

#### Estimation of standard error of the corrected genetic variance

Using Eq. (9), the se of var(**α**) can be derived with:

$$\begin{aligned} & f(var\left(\mathbf{g}\right),var\left(\varvec{\upbeta }\right),cov\left(\mathbf{g},\varvec{\upbeta }\right))=var\left(\mathbf{g}\right)-{\tau }^{2}\cdot var\left(\varvec{\upbeta }\right)\\& \quad -2\cdot \tau \cdot \left[cov\right(\mathbf{g},\varvec{\upbeta }) - \tau \cdot var(\varvec{\upbeta})], \:\text{w}\text{h}\text{e}\text{r}\text{e}\:\ {\theta }^{{\prime } }=(\frac{\partial \text{f}}{var\left(\varvec{g}\right)},\frac{\partial \text{f}}{var\left(\varvec\beta \right)},\frac{\partial \text{f}}{cov(\varvec{g},\varvec\beta )}), \end{aligned}$$

and

$$\varvec{\Omega }=\left[\begin{array}{lll}var\left(var\right(\mathbf{g}\left)\right)& cov(var\left(\mathbf{g}\right), var\left(\varvec{\upbeta }\right))& cov(var\left(\mathbf{g}\right), cor(\mathbf{g}, \varvec{\upbeta })\\ cov(var\left(\mathbf{g}\right), var\left(\varvec{\upbeta }\right))& var\left(var\left(\varvec{\upbeta }\right)\right)& cov(var\left(\varvec{\upbeta }\right),cov\left(\mathbf{g},\varvec{\upbeta }\right))\\ cov(var\left(\mathbf{g}\right), cor(\mathbf{g}, \varvec{\upbeta })& cov(var\left(\varvec{\upbeta }\right),cov\left(\mathbf{g},\varvec{\upbeta }\right))& var\left(cov\left(\mathbf{g},\varvec{\upbeta }\right)\right)\end{array}\right].$$

Again, each element of $\varvec{\Omega\:}$ can be extracted from the information matrix of the GREML method.

#### Correction of heritability

From Eqs. 6 and 10, the corrected heritability can be derived as follows, assuming **y** is standardised:

$${h}^{2}=var\left(\varvec{\upalpha\:}\right)/var\left(\mathbf{y}\right)=var\left(\varvec{\upalpha\:}\right)$$

With standardised **y**, i.e. var(**y**) = 1, the standard error of $var\left(\varvec{\upalpha\:}\right)$ can be used for the corrected heritability.

#### Correction of genetic correlation

The corrected genetic correlation due to horizontal pleiotropy is given by:

11 $${r}_{G}\:=cov(\varvec{\upalpha\:},\:\varvec{\upbeta\:})/sqrt\left(var\right(\varvec{\alpha\:}\left)\:\cdot\:var\right(\varvec{\beta\:}\left)\right).$$

Equation (11) is equivalent to:

$$\begin{aligned} {r}_{G}\:= & \:\left[cov\right(\mathbf{g},\:\varvec{\upbeta\:})\:\ -\tau\:\cdot\:var(\varvec{\upbeta\:}\left)\right]\:/\:sqrt\left(var\right(\mathbf{g})-{\tau\:}^{2}\cdot\:var(\varvec{\beta\:}) \\ & -2\cdot\:\tau\:\cdot\:[cov(\mathbf{g},\varvec{\upbeta\:})-\tau\:\cdot\:var\left(\varvec{\upbeta\:}\right)]\cdot\:var(\beta\:\left)\right) \end{aligned}$$

#### Estimation of standard error of the corrected genetic correlation

Using Eq. (9), the se of ${r}_{G}$ can be derived with

$$\begin{aligned} & f(var\left(\mathbf{g}\right),var\left(\varvec{\upbeta\:}\right),cov\left(\mathbf{g},\varvec{\upbeta\:}\right))=\left[cov\right(\mathbf{g},\:\varvec{\upbeta\:})\:\ -\:\tau\:\cdot\:var(\varvec{\upbeta\:}\left)\right]\:/\:sqrt\left(\right[var\left(\mathbf{g}\right)- \\ & \quad {\tau\:}^{2}\cdot\:var\left(\varvec{\upbeta\:}\right)-2\cdot\:\tau\:\cdot\:\left[cov\left(\mathbf{g},\varvec{\beta\:}\right)-\tau\:\cdot\:var\left(\varvec{\upbeta\:}\right)\right]\left]\:\cdot\:var\right(\varvec{\upbeta\:}\left)\right), \end{aligned}$$

$$\varvec{\theta\:}^{{\prime\:}\:}=\left(\frac{\partial\:\text{f}}{var\left(\mathbf{g}\right)},\frac{\partial\:\text{f}}{var\left(\varvec{\upbeta\:}\right)},\frac{\partial\:\text{f}}{cov(\mathbf{g},\varvec{\upbeta\:})}\right),\:\text{a}\text{n}\text{d}$$

$$\varvec{\Omega\:}=\left[\begin{array}{lll}var\left(var\right(\mathbf{g}\left)\right)&\:cov(var\left(\mathbf{g}\right),\:var\left(\varvec{\upbeta\:}\right))&\:cov(var\left(\mathbf{g}\right),\:cor(\mathbf{g},\:\varvec{\upbeta\:})\\\:cov(var\left(\mathbf{g}\right),\:var\left(\varvec{\upbeta\:}\right))&\:var\left(var\left(\varvec{\upbeta\:}\right)\right)&\:cov(var\left(\varvec{\upbeta\:}\right),cov\left(\mathbf{g},\varvec{\upbeta\:}\right))\\\:cov(var\left(\mathbf{g}\right),\:cor(\mathbf{g},\:\varvec{\upbeta\:})&\:cov(var\left(\varvec{\upbeta\:}\right),cov\left(\mathbf{g},\varvec{\upbeta\:}\right))&\:var\left(cov\left(\mathbf{g},\varvec{\upbeta\:}\right)\right)\end{array}\right].$$

Each element of $\varvec{\Omega\:}$ can be extracted from the information matrix of the GREML method.

### Binary variables

In the previous section, we derived the theoretical framework assuming that both **c** and **y** are quantitative variables. Extending this framework to binary variables, whether for **c**, **y**, or both simultaneously, requires a transformation. This transformation enables the conversion of observed-scale estimates for binary variables into estimates on the liability scale (Lee et al. 2011, 2012a, b). Although genetic correlation remains consistent between the liability and observed scales (Lee et al. 2012a, b), fixed causal effects (*τ*), phenotypic covariance (cov(y, c) in Eq. 2), heritability (*h*^*2*^), coefficient of determination (R^2^), and genetic covariance differ between these scales. Table 1 illustrates the transformation of key parameters in the HVP model between the observed and liability scale.

**Table 1:** Transformation of key parameters between the observed and liability scale

Parameter	Transformation formula
Heritability	${h}_{o}^{2}={h}_{l}^{2}\frac{{z}^{2}}{k(1-k)}$
R^2^	${R}_{o}^{2}={R}_{l}^{2}\frac{{z}^{2}}{k(1-k)}$
Genetic covariance between **c** on **y** due to horizontal pleiotropy, i.e. $\text{c}\text{o}\text{v}\left(\varvec{\upalpha\:},\varvec{\upbeta\:}\right)\:$	${\text{c}\text{o}\text{v}\left(\varvec{\upalpha\:},\varvec{\upbeta\:}\right)}_{o}={\text{c}\text{o}\text{v}\left(\varvec{\upalpha\:},\varvec{\upbeta\:}\right)}_{l}*{z}_{y}*{z}_{c}$
Genetic variance of **y**, i.e. var(**g**)	${\text{v}\text{a}\text{r}\left(\text{g}\right)}_{o}={z}_{y}^{2}*{\text{v}\text{a}\text{r}\left(\text{g}\right)}_{l}$
Causal effects of **c** on **y**	${{\uptau\:}}_{o}={{\uptau\:}}_{l}*\frac{{z}_{y}}{{z}_{c}}*\sqrt{\frac{var\left(c\right)}{var\left(y\right)}}$

For models involving binary c and/or y, transforming parameters from the observed scale (denoted by subscript o) to the liability scale (denoted by l) allows more accurate interpretations. In the transformation, we assume that the population prevalence (*k*) matches the sample prevalence, suggesting an absence of ascertainment bias. The variable z represents the height of the normal density function at a specific threshold *t* on the normal distribution. This threshold corresponds to the proportion of disease prevalence *k*, calculated as *t* = − qnorm(*k*,0,1) and *z* = dnorm(*t*,0,1) in R. For cases involving the genetic covariance cov(**α**,**β**), *z*_*y*_ is for **y** and *z*_*c*_ is for **c**.

### For summary-based data

The HVP model and parameter corrections are also applicable to summary-based data. In this approach, genetic covariance and correlation estimates obtained from LDSC can be corrected using Eqs. 8 and 11, respectively. The standard errors of these corrected estimates are derived using the block jackknife method available in LDSC ((Bulik-Sullivan et al. 2015).

### Simulation setup

Simulation was conducted using simulated genotype data. For the simulated phenotypes, 1,000 SNPs were used to model horizontal pleiotropy (with effects α_1_ and β_1_). Additionally, another 1,000 SNPs were used: half for simulating the exposure (β_2_) and the other half for simulating the outcome (α_2_), based on a sample size of 10,000 individuals. Tau is estimated based on GREML applied on the second SNP set, where the genetic effect of exposure and outcome has zero genetic covariance.

### Scenario 1: vertical Pleiotropy only

In this scenario, we simulated vertical pleiotropy only ($\tau\:$ >0), excluding horizontal pleiotropy ($cov\left(\varvec{\upalpha\:},\varvec{\upbeta\:}\right)$=0) and residual covariance ($cov\left(\mathbf{e},\varvec{\upepsilon\:}\right)$=0). The exposure (**c**) and outcome (**y**) variables were generated based on a multivariate normal distribution with a predefined variance-covariance structure, using Eq. (1). The variance-covariance structures for the genetic (α and β) and residuals (e and ε) in Eq. (1) are as follows:

$$\left[\begin{array}{ll}\text{v}\text{a}\text{r}\left(\varvec{\upalpha\:}\right)&\:cov\left(\varvec{\upalpha\:},\varvec{\upbeta\:}\right)\\\:cov\left(\varvec{\upalpha\:},\varvec{\upbeta\:}\right)&\:\text{v}\text{a}\text{r}\left(\varvec{\upbeta\:}\right)\end{array}\right]=\left[\begin{array}{ll}0.5&\:0\\\:0&\:0.5\end{array}\right]\text{a}\text{n}\text{d}$$

$$\left[\begin{array}{ll}\text{v}\text{a}\text{r}\left(\mathbf{e}\right)&\:cov\left(\mathbf{e},\varvec{\upepsilon\:}\right)\\\:cov\left(\mathbf{e},\varvec{\upepsilon\:}\right)&\:\text{v}\text{a}\text{r}\left(\varvec{\upepsilon\:}\right)\end{array}\right]=\left[\begin{array}{ll}1-\text{v}\text{a}\text{r}\left(\varvec{\upalpha\:}\right)-{\tau\:}^{2}-2\tau\:cov\left(\varvec{\upalpha\:},\varvec{\upbeta\:}\right)&\:0\\\:0&\:0.5\end{array}\right],$$

where $$\text{v}\text{a}\text{r}\left(\mathbf{e}\right)=1-\text{v}\text{a}\text{r}$$ $$\left(\varvec{\upalpha\:}\right)-{\tau\:}^{2}-2\tau\:cov$$$$\left(\varvec{\upalpha\:},\varvec{\upbeta\:}\right)$$ is used to maintain the phenotypic variance of **y** equal to 1.

This setup results in a heritability of 0.5 for both **c** and **y**. In this scenario, we systematically varied the causal effect *τ* from 0 to 0.4 in increments of 0.1. For genetic data, we simulated genotypes for 1000 SNPs using a binomial distribution with a minor allele frequency (MAF) set at 0.2.

### Scenario 2: both vertical and horizontal Pleiotropy

In the second scenario, we introduced a more intricate setting involving both vertical and horizontal pleiotropy ($\tau\:$ >0 and cov(**α**,**β**) >0), while still excluding residual covariance (cov(**e**,**ε**)=0). To delineate between vertical and horizontal pleiotropy variants, two sets of SNPs were exclusively used, each comprising 1000 SNPs, contributing to distinct genetic effects for **y** and **c**. The genetic effects can be decomposed as follows: **α** = **α₁** + **α₂** and **β** = **β₁** + **β₂**. The variance-covariance structures for the genetic effects (**α₁** and **β₁**, and **α₂** and **β₂**) are as follows:

$$\left[\begin{array}{ll}\text{v}\text{a}\text{r}\left({\varvec{\upalpha\:}}_{1}\right)&\:cov\left({\varvec{\upalpha\:}}_{1},{\varvec{\upbeta\:}}_{1}\right)\\\:cov\left({\varvec{\upalpha\:}}_{1},{\varvec{\upbeta\:}}_{1}\right)&\:\text{v}\text{a}\text{r}\left({\varvec{\upbeta\:}}_{1}\right)\end{array}\right]=\left[\begin{array}{ll}0.35&\:0.25\\\:0.25&\:0.26\end{array}\right]$$

and

$$\left[\begin{array}{ll}\text{v}\text{a}\text{r}\left({\varvec{\upalpha\:}}_{2}\right)&\:cov\left({\varvec{\upalpha\:}}_{2},{\varvec{\upbeta\:}}_{2}\right)\\\:cov\left({\varvec{\upalpha\:}}_{2},{\varvec{\upbeta\:}}_{2}\right)&\:\text{v}\text{a}\text{r}\left({\varvec{\upbeta\:}}_{2}\right)\end{array}\right]=\left[\begin{array}{ll}0.15&\:0\\\:0&\:0.24\end{array}\right].$$

The variance-covariance structure for the residual effects (e and ε) is as follows:

$$\begin{aligned} & \left[\begin{array}{ll}\text{v}\text{a}\text{r}\left(\mathbf{e}\right)&\:cov\left(\mathbf{e},\varvec{\upepsilon\:}\right)\\\:cov\left(\mathbf{e},\varvec{\upepsilon\:}\right)&\:\text{v}\text{a}\text{r}\left(\varvec{\upepsilon\:}\right)\end{array}\right] \\ & \quad =\left[\begin{array}{ll}1-\text{v}\text{a}\text{r}({\varvec{\upalpha\:}}_{1}+{\varvec{\upalpha\:}}_{2})-{\tau\:}^{2}-2\tau\:cov\left({\varvec{\upalpha\:}}_{1}+{\varvec{\upalpha\:}}_{2},{\varvec{\upbeta\:}}_{1}+{\varvec{\upbeta\:}}_{2}\right)&\:0\\\:0&\:0.5\end{array}\right]. \end{aligned}$$

where $$\text{v}\text{a}\text{r}\left(\mathbf{e}\right)=1-\text{v}\text{a}\text{r}({\varvec{\upalpha\:}}_{1}+{\varvec{\upalpha\:}}_{2})$$$$-{\tau\:}^{2}-2\tau\:cov$$$$({\varvec{\upalpha\:}}_{1}+{\varvec{\upalpha\:}}_{2},$$$${\varvec{\upbeta\:}}_{1}+{\varvec{\upbeta\:}}_{2})$$ is used to maintain the phenotypic variance of **y** equal to 1.

This setup results in a heritability of 0.5 for both **c** and **y**, and genetic correlation attributed to horizontal pleiotropy is 0.5. In this scenario, we systematically varied the causal effect *τ* from 0 to 0.4 in increments of 0.1.

### Scenario 3: vertical and horizontal Pleiotropy plus residual covariance

In the third scenario, we introduced a more complex setting involving both vertical and horizontal pleiotropy ($\tau\:$ >0 and *cov*(**α**,**β**) >0), along with the inclusion of residual covariance (*cov*(**e**,**ε**)>0). Similar to scenario 2, we used two distinct sets of SNPs, each consisting of 1000 SNPs, to distinguish between vertical and horizontal pleiotropy. The variance-covariance structures for the genetic effects (**α₁** and **β₁**, and **α₂** and **β₂**) are as follows:

$$\left[\begin{array}{ll}\text{v}\text{a}\text{r}\left({\varvec{\upalpha\:}}_{1}\right)&\:cov\left({\varvec{\upalpha\:}}_{1},{\varvec{\upbeta\:}}_{1}\right)\\\:cov\left({\varvec{\upalpha\:}}_{1},{\varvec{\upbeta\:}}_{1}\right)&\:\text{v}\text{a}\text{r}\left({\varvec{\upbeta\:}}_{1}\right)\end{array}\right]=\left[\begin{array}{ll}0.35&\:0.25\\\:0.25&\:0.26\end{array}\right]$$

and

$$\left[\begin{array}{ll}\text{v}\text{a}\text{r}\left({\varvec{\upalpha\:}}_{2}\right)&\:cov\left({\varvec{\upalpha\:}}_{2},{\varvec{\upbeta\:}}_{2}\right)\\\:cov\left({\varvec{\upalpha\:}}_{2},{\varvec{\upbeta\:}}_{2}\right)&\:\text{v}\text{a}\text{r}\left({\varvec{\upbeta\:}}_{2}\right)\end{array}\right]=\left[\begin{array}{ll}0.15&\:0\\\:0&\:0.24\end{array}\right].$$

The variance-covariance structure for the residual effects (**e** and **ε**) is as follows:

$$\begin{aligned} & \left[\begin{array}{ll}\text{v}\text{a}\text{r}\left(\mathbf{e}\right)& \:cov\left(\mathbf{e},\varvec{\upepsilon\:}\right)\\\:cov\left(\mathbf{e},\varvec{\upepsilon\:}\right)& \:\text{v}\text{a}\text{r}\left(\varvec{\upepsilon\:}\right)\end{array}\right] \\ & \quad = \left[\begin{array}{ll} {\begin{aligned} & 1-\text{v}\text{a}\text{r}({\varvec{\upalpha\:}}_{1}+{\varvec{\upalpha\:}}_{2})-{\tau\:}^{2} -2\tau\:cov\left({\varvec{\upalpha\:}}_{1}+{\varvec{\upalpha\:}}_{2},{\varvec{\upbeta\:}}_{1}+{\varvec{\upbeta\:}}_{2}\right)\\ & \quad-2\tau\:cov\left(\text{e},{\upepsilon\:}\right) \end{aligned}}&\:0.1\\\:0.1&\:0.5\end{array}\right]. \end{aligned}$$

where $$\text{v}\text{a}\text{r}\left(\mathbf{e}\right)=1-\text{v}\text{a}\text{r}({\varvec{\upalpha\:}}_{1}+{\varvec{\upalpha\:}}_{2})-{\tau\:}^{2}-2\tau\:cov$$$$({\varvec{\upalpha\:}}_{1} +{\varvec{\upalpha\:}}_{2}, $$$${\varvec{\upbeta\:}}_{1}+{\varvec{\upbeta\:}}_{2})-2\tau\:cov\left(\mathbf{e},\varvec{\upepsilon\:}\right) $$ is used to maintain the phenotypic variance of **y** equal to 1.

This setup results in a heritability of 0.5 for both **c** and **y**, and genetic correlation attributed to horizontal pleiotropy is 0.5, and fixed residual correlation at 0.1. In this scenario, we systematically varied the causal effect *τ* from 0 to 0.4 in increments of 0.1.

### Scenario 4: complete mediation of genetic effect of trait 1(y)

In this simulation scenario, we aimed to explore the complete mediation of genetic effects on trait **y** (var(**α**) = 0) through another intermediate trait, **c**, which exhibits significant direct genetic effects (var(**β**) = 0.5). Consequently, we set the covariance between **α** and **β** to 0 (*cov*(**α**, **β**) = 0), ensuring that no direct genetic effects influence **y** without passing through **c**. Additionally, we exclude residual covariance (*cov*(**e**, **ε**) = 0) to focus exclusively on vertical pleiotropy (*τ* > 0). Our interest lay in understanding how genetic effects manifest via c, acting as the primary genetic influencers on **y** in the presence of vertical pleiotropy ($\tau\:$ >0). We constructed the exposure (**c**) and outcome (**y**) variables using a multivariate normal distribution, defining their variance-covariance structure according to Eq. (1). The variance-covariance structures for the genetic (**α** and **β**) and residuals (**e** and **ε**) are represented as follows:

$$\left[\begin{array}{ll}\text{v}\text{a}\text{r}\left(\varvec{\upalpha\:}\right)&\:cov\left(\varvec{\upalpha\:},\varvec{\upbeta\:}\right)\\\:cov\left(\varvec{\upalpha\:},\varvec{\upbeta\:}\right)&\:\text{v}\text{a}\text{r}\left(\varvec{\upbeta\:}\right)\end{array}\right]=\left[\begin{array}{ll}0&\:0\\\:0&\:0.5\end{array}\right]$$

and

$$\left[\begin{array}{ll}\text{v}\text{a}\text{r}\left(\mathbf{e}\right)&\:cov\left(\mathbf{e},\varvec{\upepsilon\:}\right)\\\:cov\left(\mathbf{e},\varvec{\upepsilon\:}\right)&\:\text{v}\text{a}\text{r}\left(\varvec{\upepsilon\:}\right)\end{array}\right]=\left[\begin{array}{ll}1-{\tau\:}^{2}&\:0\\\:0&\:0.5\end{array}\right].$$

where $\text{v}\text{a}\text{r}\left(\mathbf{e}\right)=1-{\tau\:}^{2}\:$is used to maintain the phenotypic variance of **y** equal to 1. In this setup, the heritability for **c** is 0.5, while it is 0 for **y**, with a genetic correlation attributed solely to vertical pleiotropy. The scenario systematically varied the causal effect *τ* from 0 to 0.4 in increments of 0.1, elucidating the extent of genetic mediation through trait **c** on the outcome **y**.

### Scenario 5: vertical and horizontal Pleiotropy for the exposure, but only horizontal Pleiotropy for the outcome

The fifth scenario is a special form of scenario 2 where the causal SNPs for **y** include only horizontal pleiotropy, with no vertical pleiotropy (*var*(**α₂**) = 0). The variance-covariance structures for the genetic effects (**α₁** and **β₁**, and **α₂** and **β₂**) are as follows:

$$\left[\begin{array}{ll}\text{v}\text{a}\text{r}\left({\varvec{\upalpha\:}}_{1}\right)&\:cov\left({\varvec{\upalpha\:}}_{1},{\varvec{\upbeta\:}}_{1}\right)\\\:cov\left({\varvec{\upalpha\:}}_{1},{\varvec{\upbeta\:}}_{1}\right)&\:\text{v}\text{a}\text{r}\left({\varvec{\upbeta\:}}_{1}\right)\end{array}\right]=\left[\begin{array}{ll}0.5&\:0.25\\\:0.25&\:0.26\end{array}\right]$$

and

$$\left[\begin{array}{ll}\text{v}\text{a}\text{r}\left({\varvec{\upalpha\:}}_{2}\right)&\:cov\left({\varvec{\upalpha\:}}_{2},{\varvec{\upbeta\:}}_{2}\right)\\\:cov\left({\varvec{\upalpha\:}}_{2},{\varvec{\upbeta\:}}_{2}\right)&\:\text{v}\text{a}\text{r}\left({\varvec{\upbeta\:}}_{2}\right)\end{array}\right]=\left[\begin{array}{ll}0&\:0\\\:0&\:0.24\end{array}\right].$$

The variance-covariance structure for the residual effects (**e** and **ε**) is as follows:

$$\begin{aligned} \left[\begin{array}{ll}\text{v}\text{a}\text{r}\left(\mathbf{e}\right)&\:cov\left(\mathbf{e},\varvec{\upepsilon\:}\right)\\\:cov\left(\mathbf{e},\varvec{\upepsilon\:}\right)&\:\text{v}\text{a}\text{r}\left(\varvec{\upepsilon\:}\right)\end{array}\right]=\left[\begin{array}{ll} {\begin{aligned} & 1-\text{v}\text{a}\text{r}\left({\varvec{\upalpha\:}}_{1}\right)-{\tau\:}^{2} \\ & \quad -2\tau\:cov\left({\varvec{\upalpha\:}}_{1},{\varvec{\upbeta\:}}_{1}+{\varvec{\upbeta\:}}_{2}\right) \end{aligned}}&\:0\\\:0&\:0.5\end{array}\right]. \end{aligned}$$

where $$\text{v}\text{a}\text{r}\left(\mathbf{e}\right)$$$$=1-\text{v}\text{a}\text{r}\left({\varvec{\upalpha\:}}_{1}\right)$$$$-{\tau\:}^{2}-2\tau\:cov({\varvec{\upalpha\:}}_{1},$$$${\varvec{\upbeta\:}}_{1}$$$$+{\varvec{\upbeta\:}}_{2})$$ is used to maintain the phenotypic variance of **y** equal to 1.

This setup results in a heritability of 0.5 for both **c** and **y**, and genetic correlation attributed to horizontal pleiotropy is 0.5. In this scenario, we systematically varied the causal effect *τ* from 0 to 0.4 in increments of 0.1.

We also performed simulations based on real genotype data from the UKB with MAF > 0.01. Details of the UKB genotype-based simulation are provided in the Supplementary Note.

### Simulation for binary outcome and exposure

In real situations, both outcomes and exposures can often be binary. To simulate binary outcomes or exposures, we used the liability threshold model, which is based on predefined population prevalence rates (k). First, continuous quantitative phenotypes were simulated as described earlier. Then, using the specified prevalence rates for the outcome (**y)** and exposure (**c)**, we applied standard normal distribution theory to determine the corresponding liability thresholds. Individuals were then categorized as either having or not having the outcome, or as exposed or unexposed, depending on whether their continuous phenotypes exceeded the respective threshold values.

### Application of real data: MetS comorbidities and related complex traits

#### Phenotype data

We investigated MetS and 23 associated traits— 12 comorbidities and 11 quantitative traits— to explore their biological interrelationships. MetS was defined as a composite phenotype according to the International Diabetes Federation criteria (Alberti et al. 2009). Individuals were classified as having MetS if they met three or more of the following criteria: central obesity (waist circumference (WC) ≥ 88 cm for females and ≥ 102 cm for males), elevated fasting triglycerides (TG) (≥ 1.7 mmol/L or medication), reduced high-density lipoprotein cholesterol (HDL-C) (< 1.29 mmol/L for females and < 1.03 mmol/L for males or medication), elevated blood pressure (systolic/diastolic blood pressure [SBP/DBP] ≥ 130/85 mmHg), and elevated fasting glucose (GLU) (≥ 5.6 mmol/L or medication). The 12 comorbidities were identified using ICD-10 codes (Supplementary Table 1), with relevant conditions from chapters including cardiovascular, psychiatric, metabolic, respiratory, and renal disorders. Quantitative traits included body mass index (BMI), basal metabolic rate (BMR), lung function, insulin-like growth factor 1 (IGF-1), neuroticism, C-reactive protein (CRP), serum vitamin D, and liver function tests (Alkaline Phosphatase (ALP), Alanine Aminotransferase (ALT), Aspartate Aminotransferase (ASP), & Gamma-Glutamyl Transferase (GGT) (Supplementary Table 2).

Data were adjusted for confounders (age, sex, Townsend Deprivation Index (TDI), education level, assessment centre, batch effect, population structure using the first 10 principal components), with standardized residuals used in analyses.

#### Genotype data

Rigorous quality control procedures were applied using PLINK v1.9. Individuals were excluded if they did not self-identify as white British, had discordant sex information, or had a genotype missing rate > 0.05. At the variant level, exclusions were made for an INFO score < 0.6, minor allele frequency < 0.01, Hardy-Weinberg equilibrium p-value < 1E-7, or call rate < 0.95. A total of 288,792 individuals and 7,701,772 single nucleotide polymorphisms (SNPs) passed these quality controls. Given the computational demands when using individual-level data, a random sample of 82,955 participants and HapMap3 SNPs was selected to balance computational efficiency with sufficient statistical power. The remaining 205,828 samples were used for two sample MR analysis of exposure GWAS, as detailed below.

#### Genetic correlation

We estimated genetic correlations between MetS and its associated traits and comorbidities using bivariate GREML, a method that utilizes individual-level data to quantify the genetic overlap between traits. To account for multiple testing, we applied a Bonferroni correction to adjust significance thresholds, minimizing the risk of false-positive findings.

Subsequently, GWAS was conducted on the same dataset to generate summary statistics, followed by LDSC to estimate genetic correlations from these summary data. To further refine the genetic correlations, we applied HVP to disentangle horizontal and vertical pleiotropy, incorporating causal relationships between traits as estimated through MR. This process enabled a direct comparison of corrected genetic correlations derived from individual-level GREML estimates and summary-based LDSC estimates, offering an additional layer of validation and robustness to our findings.

To estimate causal effects, two-sample MR was conducted using the “TwoSampleMR” R package where a separate sample (*n* = 205,837) was used for exposure GWAS. Methods included MR Egger regression (Bowden et al. 2015), Inverse Variance Weighting (IVW), weighted median (Bowden et al. 2016), and weighted mode (Bowden et al. 2019). Additionally, GSMR in GCTA(v1.93) (Zhu et al. 2018), the contamination mixture method (Burgess et al. 2020), Iterative Mendelian Randomization and Pleiotropy (IMRP) (Zhu et al. 2021), Latent outcome variable approach (MRLOVA) (Amente et al. 2025) and The MR Pleiotropy RESidual Sum and Outlier (MR-PRESSO) (Verbanck et al. 2018) method were also employed to address significant heterogeneity and horizontal pleiotropy. Instrumental SNPs were selected based on genome-wide significance, LD clumped and harmonized between exposure and outcome datasets. Sensitivity tests evaluated directional pleiotropy, heterogeneity, and the strength of instrumental variables using F-statistics.

## Results

### Simulations

**Fig. 2:**
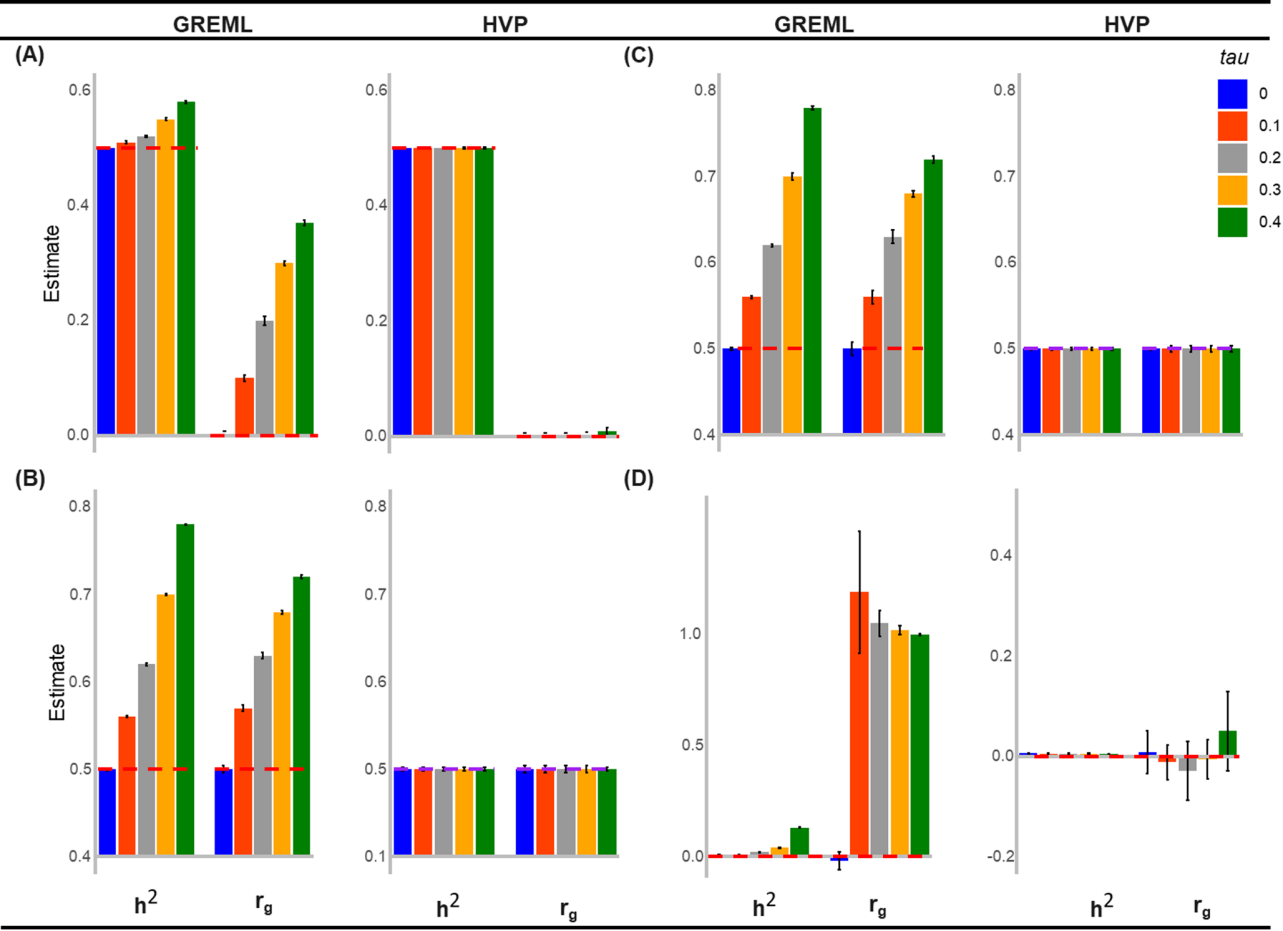
Estimated Heritability (h^2^) and Genetic correlation (r_g_). This figure presents results from a simulation examining the heritability of trait **y** and the genetic correlation of traits **y** and **c** under four scenarios. The red dashed lines represent the true values. **A** (scenario 1): Traits **y** and **c** are simulated under vertical pleiotropy only, with variance-covariance structures for genetic effects $$\left[\begin{array}{ll}var\left(\varvec{\upalpha\:}\right)&\:cov\left(\varvec{\upalpha\:},\varvec{\upbeta\:}\right)\\\:cov\left(\varvec{\upalpha\:},\varvec{\upbeta\:}\right)&\:var\left(\varvec{\upbeta\:}\right)\end{array}\right]$$$$=\left[\begin{array}{ll}0.5&\:0\\\:0&\:0.5\end{array}\right]$$ and residual effects $$\left[\begin{array}{ll}var\left(\mathbf{e}\right)&\:cov\left(\mathbf{e},\varvec{\upepsilon\:}\right)\\\:cov\left(\mathbf{e},\varvec{\upepsilon\:}\right)&\:var\left(\varvec{\upepsilon\:}\right)\end{array}\right]$$ $$=\left[\begin{array}{ll}1-var\left(\varvec{\upalpha\:}\right)-{\tau\:}^{2}-2\tau\:cov\left(\varvec{\upalpha\:},\varvec{\upbeta\:}\right)&\:0\\\:0&\:0.5\end{array}\right]$$ maintain **y**’s phenotypic variance at 1. The left pane shows the biased estimates due to vertical pleiotropy. The right panel demonstrates that, after estimating τ, the HPV model successfully disentangle horizontal pleiotropy from the vertical pleiotropy, thereby correcting both heritability and genetic correlation estimates. **B** (scenario 2): Traits **y** and **c** are simulated under both horizontal and vertical pleiotropy without residual covariance. The variance-covariance structures for genetic effects $$\left[\begin{array}{ll}var\left({\varvec{\upalpha\:}}_{1}\right)&\:cov\left({\varvec{\upalpha\:}}_{1},{\varvec{\upbeta\:}}_{1}\right)\\\:cov\left({\varvec{\upalpha\:}}_{1},{\varvec{\upbeta\:}}_{1}\right)&\:var\left({\varvec{\upbeta\:}}_{1}\right)\end{array}\right]=\left[\begin{array}{ll}0.35&\:0.25\\\:0.25&\:0.26\end{array}\right]$$ and $$\left[\begin{array}{ll}var\left({\varvec{\upalpha\:}}_{2}\right)&\:cov\left({\varvec{\upalpha\:}}_{2},{\varvec{\upbeta\:}}_{2}\right)\\\:cov\left({\varvec{\upalpha\:}}_{2},{\varvec{\upbeta\:}}_{2}\right)&\:var\left({\varvec{\upbeta\:}}_{2}\right)\end{array}\right]=\left[\begin{array}{ll}0.15&\:0\\\:0&\:0.24\end{array}\right]$$. Residual effects (**e** and **ε**) are characterized by $$\left[\begin{array}{ll}var\left(\mathbf{e}\right)&\:0\\\:0&\:0.5\end{array}\right]$$, maintaining **y**’s phenotypic variance at 1. The left pane shows the biased estimates due to vertical pleiotropy. The right panel demonstrates that, after estimating τ, the HPV model successfully disentangle horizontal pleiotropy from the vertical pleiotropy, thereby correcting both heritability and genetic correlation estimates. **C** (scenario 3): Traits **y** and **c** are simulated under both horizontal and vertical pleiotropy, with residual covariance. The variance-covariance structures for genetic effects are $$\left[\begin{array}{ll}var\left({\varvec{\upalpha\:}}_{1}\right)&\:cov\left({\varvec{\upalpha\:}}_{1},{\varvec{\upbeta\:}}_{1}\right)\\\:cov\left({\varvec{\upalpha\:}}_{1},{\varvec{\upbeta\:}}_{1}\right)&\:var\left({\varvec{\upbeta\:}}_{1}\right)\end{array}\right]=\left[\begin{array}{ll}0.35&\:0.25\\\:0.25&\:0.26\end{array}\right]$$ and $$\left[\begin{array}{ll}var\left({\varvec{\upalpha\:}}_{2}\right)&\:cov\left({\varvec{\upalpha\:}}_{2},{\varvec{\upbeta\:}}_{2}\right)\\\:cov\left({\varvec{\upalpha\:}}_{2},{\varvec{\upbeta\:}}_{2}\right)&\:var\left({\varvec{\upbeta\:}}_{2}\right)\end{array}\right]=\left[\begin{array}{ll}0.15&\:0\\\:0&\:0.24\end{array}\right]$$. Residual effects (**e** and **ε**) are characterized by $$\left[\begin{array}{ll}var\left(\mathbf{e}\right)&\:0.1\\\:0.1&\:0.5\end{array}\right]$$, maintaining **y**’s phenotypic variance at 1. The left pane shows the biased estimates due to vertical pleiotropy. The right panel demonstrates that, after estimating τ, the HPV model successfully disentangle horizontal pleiotropy from the vertical pleiotropy, thereby correcting both heritability and genetic correlation estimates. **D** (scenario 4): The heritability estimation for trait **y** and the genetic correlation between traits **y** and **c**, simulated under the assumption of no direct genetic effect on trait **y** except through trait **c** (complete mediation of genetic effects via the exposure). The variance-covariance structures for genetic effects $$\left[\begin{array}{ll}var\left(\varvec{\upalpha\:}\right)&\:cov\left(\varvec{\upalpha\:},\varvec{\upbeta\:}\right)\\\:cov\left(\varvec{\upalpha\:},\varvec{\upbeta\:}\right)&\:var\left(\varvec{\upbeta\:}\right)\end{array}\right]=\left[\begin{array}{ll}0&\:0\\\:0&\:0.5\end{array}\right]$$ and residual effects$$\:\left[\begin{array}{ll}var\left(\mathbf{e}\right)&\:cov\left(\mathbf{e},\varvec{\upepsilon\:}\right)\\\:cov\left(\mathbf{e},\varvec{\upepsilon\:}\right)&\:var\left(\varvec{\upepsilon\:}\right)\end{array}\right]=\left[\begin{array}{ll}1-{\tau\:}^{2}&\:0\\\:0&\:0.5\end{array}\right]$$ maintain **y**’s phenotypic variance at 1. The left pane shows the biased estimates due to vertical pleiotropy. The right panel demonstrates that, after estimating τ, the HPV model successfully disentangle horizontal pleiotropy from the vertical pleiotropy, thereby correcting both heritability and genetic correlation estimates

The HVP model integrates both horizontal and vertical pleiotropy. In this model, vertical pleiotropy mediates the genetic effects on the outcome (see Fig. 1). As demonstrated in Eqs. (5–7), vertical pleiotropy can introduce biases into estimates of genetic variances within a univariate context. These biases extend to genetic covariances, impacting key genetic parameters such as heritability and genetic correlation (refer to Figs. 2, Supplementary Fig. 1). Through extensive simulation analyses (scenarios 1–5), we have identified biases in estimating SNP-based heritability and genetic correlation using the existing method, bivariate GREML (as depicted in Eq. (5)).

For instance, in simulation scenario 1, where there is no shared genetic effect between two phenotypes (*cov*(**α**,**β**) = 0), varying the fixed causal effect τ from 0.0 to 0.4 in increments of 0.1 reveals a significant deviation in SNP-based heritability and genetic correlation. Notably, this deviation is proportional to the magnitude of $\tau\:$ (refer to Fig. 2A). When applying the proposed method (Eq. (6)– Eq. (11)), the biased estimates can be corrected, and unbiased estimates for both heritability and genetic correlations can be obtained (Fig. 2A).

A similar pattern was observed when using simulation scenarios 2–4, where genetic and residual correlations are presented (Figs. 2B-D), in that the existing method generates biased estimates that can be corrected by our proposed method. We also conducted simulations (scenario 4) where genetics has no direct effect on the outcome that is causally associated with another trait (complete mediation of genetic effect via the second trait). Interestingly, because of this causal relationship, the non-heritable trait (outcome) becomes a heritable trait (Fig. 2D (the left panel)). This bias can be also corrected by the proposed method (Fig. 2D (the right panel)). Also, similar pattern was observed under the fifth Scenario (Supplementary Fig. 2).

Our extensive simulations demonstrate that our approach effectively disentangles the two components of pleiotropy across scenarios 1 through 4 (Fig. 2). Simulations based on real UK Biobank (UKB) genotype data yielded similar results, underscoring the robustness of our method (see Supplementary Fig. 1). Additionally, we verified the applicability of the HVP model and parameter corrections for binary exposure and/or binary outcome using the transformation approach (see Supplementary Table 3).

The HVP model also corrects the underestimation of genetic correlation and outcome heritability in scenarios where the causal effect (τ) is negative (supplementary Tables 4–5).

### Real data

#### MetS comorbidities and related complex traits

From a total of 82,955 white British included in this analysis, a total of 17,335 (20.90%) were identified as MetS cases at baseline (Supplementary Table 6). Heritability of complex traits evaluated in this study ranges from 29.18% for BMR to 0.77% for stroke (Supplementary Table 7). We examined the prevalence of ICD-10 codes associated with MetS in individuals with (*n* = 17,335) and without MetS (*n* = 65,620). All comorbidities were more frequent in the MetS group, with significant differences confirmed by chi-square tests after multiple test correction. After adjusting for covariates, the associations of stroke and atrial fibrillation with MetS were no longer significant, while others remained significant (Supplementary Table 8). Additionally, baseline comparisons of the quantitative traits showed significant mean differences between the groups, persisting after adjustment except for the FEV1/FVC Z-score and neuroticism (Supplementary Table 9).

#### Estimation of causal relationship

**Fig. 3:**
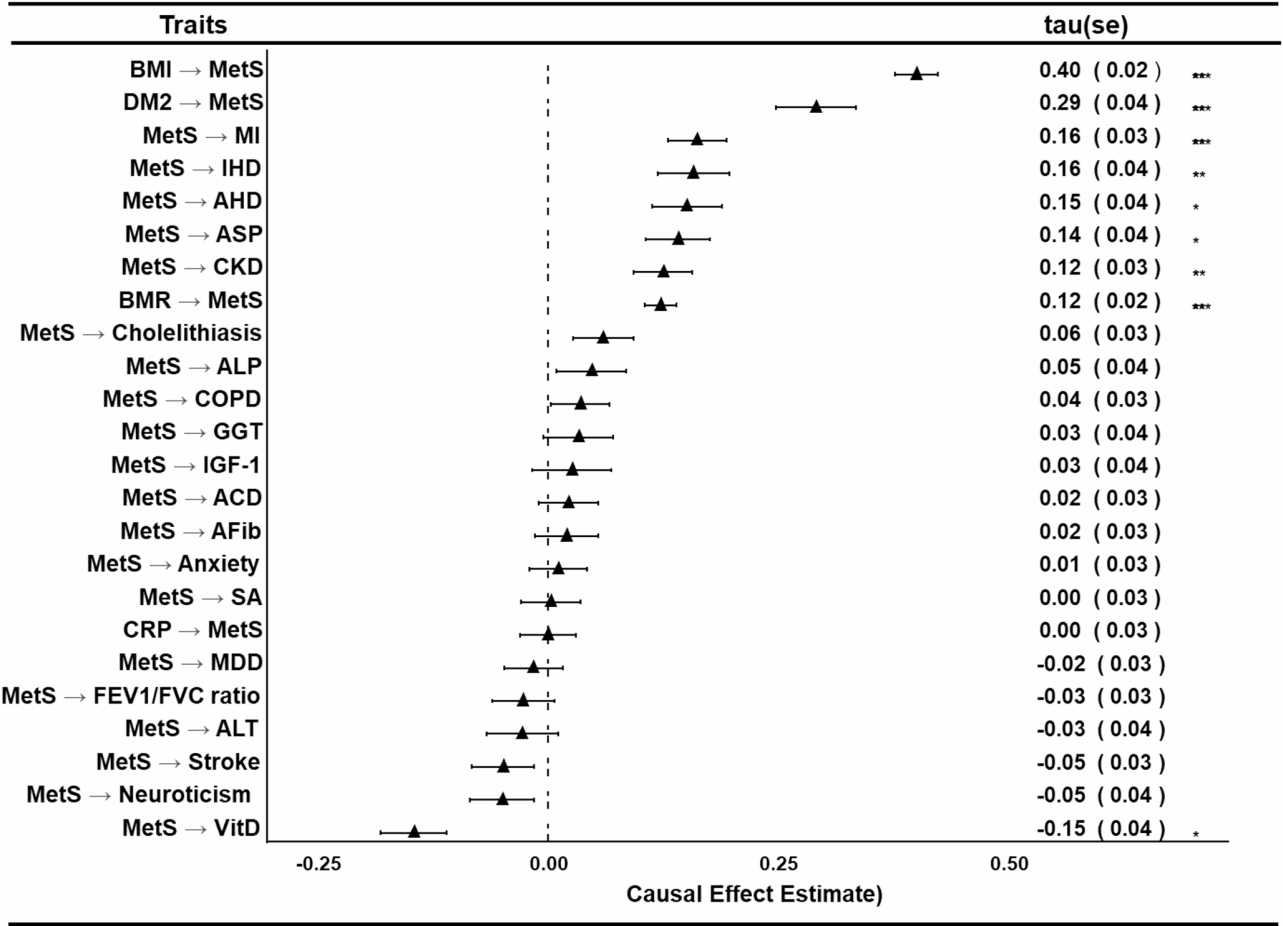
Causal relationships between MetS and related complex traits. Causal effects estimates determined by MRLOVA, a robust method, are presented here for clarity and focus. Consistent findings across other methods (detailed in Supplementary Figs. 2 & 4) further validate and strengthen the robustness of these results. MetS metabolic syndrome, AHD atherosclerotic heart disease, IHD ischemic heart disease, MI myocardial infarction, AFib atrial fibrillation, ACD arrhythmia and conduction disorders, CKD chronic kidney disease, DM2 type 2 diabetes, SA sleep apnoea, BMI body mass index, BMR basal metabolic rate, IGF-1 insulin-like growth factor 1, CRP C-reactive protein, vitD vitamin D, ALP Alkaline Phosphatase, ALT Alanine Aminotransferase, ASP Aspartate Aminotransferase, & GGT Gamma-Glutamyl Transferase

In the analysis of causal relationships, MetS was examined as both an exposure and an outcome for traits of interest associated with MetS (Figs. 3, Supplementary Figs. 3 and 5, Supplementary Table 10). Based on prior knowledge and current literature, MetS was primarily analyzed as an exposure (Lin et al. 2022; Mottillo et al. 2010; Zhu et al. 2023) for most comorbidities and complex traits, while it was considered an outcome in analyses involving BMI, BMR, CRP, and vitamin D (Xu et al. 2020; Ridker et al. 2008; Timpson et al. 2005). In all cases, reverse causation was systematically evaluated to ensure robustness of the findings (Supplementary Figs. 4 and 6). Instrumental variant details, including F-statistics, are in Supplementary Table 11.

When analyzing MetS as an outcome, genetically determined BMI and BMR were positively associated with MetS status. This association was consistent across all methods, except for the weighted mode, which reported a non-significant association for BMR (Fig. 3, Supplementary Fig. 3, Supplementary Table 10). No evidence of reverse causation was detected (Supplementary Fig. 4). No causal relationship was identified between genetically determined serum CRP levels and MetS (Fig. 3, Supplementary Fig. 3), nor between MetS and CRP levels, also confirming the absence of reverse causation (Supplementary Fig. 4). Genetically determined vitamin D levels also showed no significant effect on MetS. However, MetS exhibited a significant negative impact on vitamin D levels, with this reverse causal effect consistently supported by multiple methods, including GSMR, MR-PRESSO, ConMix, and MRLOVA (Fig. 3, Supplementary Figs. 3–4, Supplementary Table 10).

When analyzing MetS as an exposure, genetically determined MetS was significantly associated with the development of cardiovascular diseases, including myocardial infarction (MI), ischemic heart disease (IHD), and atherosclerotic heart disease (AHD) (Fig. 3, Supplementary Fig. 5, Supplementary Table 10). Estimates from all methods, except MR Egger, were consistent in direction and magnitude, with no evidence of reverse causation between cardiovascular diseases and MetS (Supplementary Fig. 6). Genetically determined MetS was also significantly associated with chronic kidney disease (CKD) and type 2 diabetes (DM2). While no reverse causation was detected for CKD, a consistent bidirectional relationship between MetS and DM2 was observed across all methods except MR Egger (Supplementary Figs. 5–6, Supplementary Table 10). No causal association was identified between MetS and atrial fibrillation (AFib) in either direction (Fig. 3, Supplementary Fig. 5–6, Supplementary Table 10).

#### Genetic correlation estimations

**Fig. 4:**
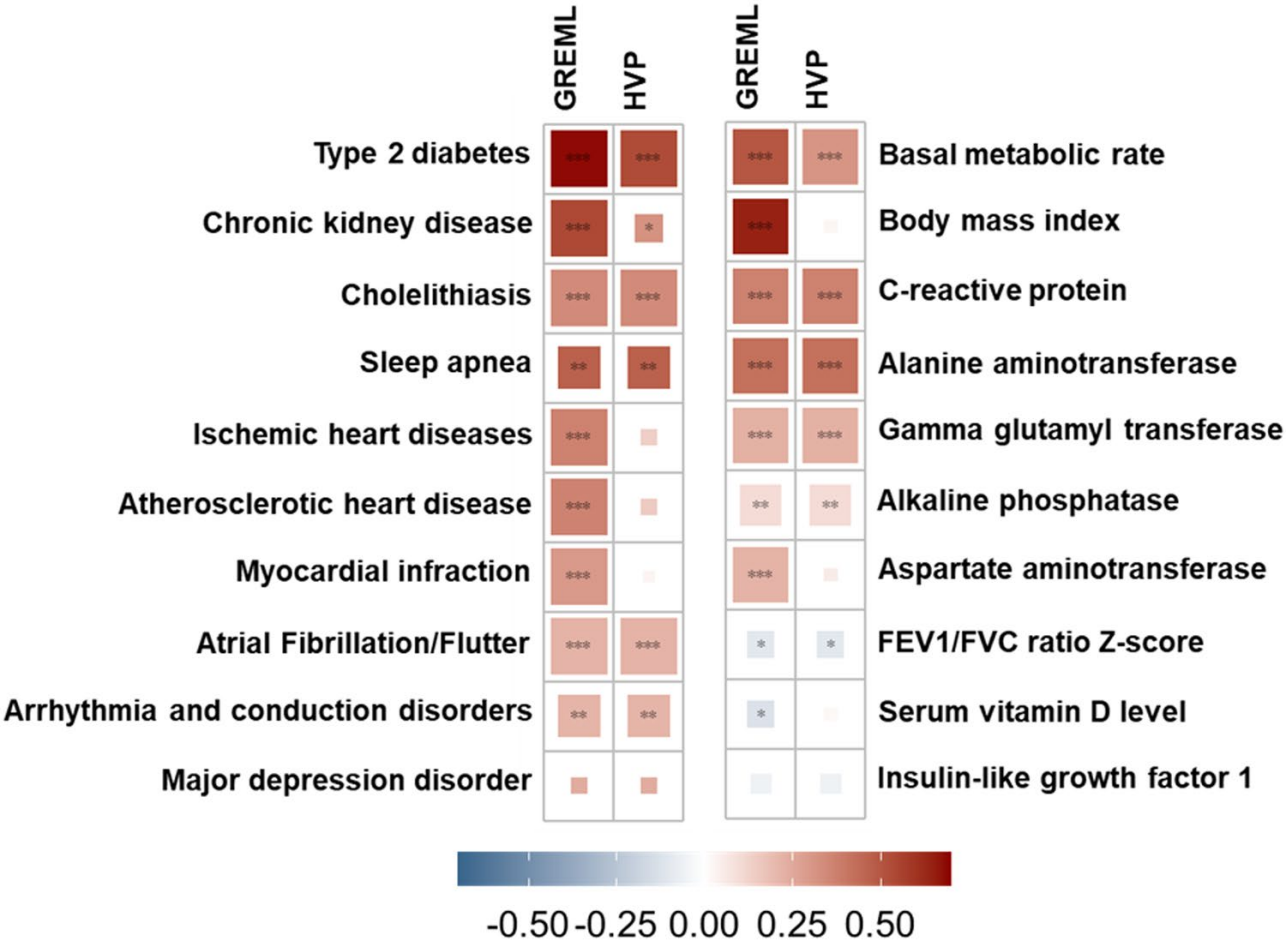
Genetic correlation between MetS and related traits estimated using conventional GREML and the proposed HVP methods. Bonferroni correction for multiple testing were applied a to adjust significance thresholds. Causal effect estimated by MRLOVA is used to correct the genetic correlation in this figure

The genetic correlation between MetS and 23 complex traits (including 12 comorbid conditions based on ICD-10 and 11 quantitative traits) was estimated using the UKB data. All phenotypes were adjusted for confounders such as age, sex, and other relevant factors. We applied the conventional bivariate GREML method (Ni et al. 2018) for this analysis. The strongest genetic correlation was observed between MetS and type 2 diabetes (0.69, se = 0.0367), followed by BMI (0.65 se = 0.019), CKD (0.54, se = 0.0843), and BMR (0.50, se = 0.0196). Serum vitamin D levels exhibited a negative correlation with MetS (-0.12, se = 0.0358), as did IGF-1 (-0.06, se = 0.0261). Notably, stroke, neuroticism, chronic obstructive pulmonary disease (COPD), and anxiety showed no significant genetic correlations with MetS (see Fig. 4, Supplementary Table 10).

Based on these estimations, we applied the proposed HVP model to correct the genetic correlations and disentangle the effects of horizontal pleiotropy. The estimated genetic correlations between MetS and cardiovascular diseases were primarily driven by vertical pleiotropy, as evidenced by the significant causal effects of MetS on cardiovascular diseases. After correcting using the HVP model, the genetic correlation attributed to horizontal pleiotropy became non-significant. Similarly, the genetic correlation between vitamin D and MetS became non-significant after correction, suggesting no significant horizontal pleiotropy effects (Fig. 4).

Interestingly, the strong genetic correlation between DM2 and MetS was influenced by both vertical and horizontal pleiotropy, with horizontal pleiotropy accounting for 77.67% of the correlation i.e., the proportion of the original genetic correlation that remains after adjusting for vertical pleiotropy. Likewise, the genetic correlation between BMR and MetS was also affected by both forms of pleiotropy. The corrected genetic correlation between MetS and CKD had a p-value of 0.0023 but became non-significant after adjusting for multiple testing. In contrast, the genetic correlations between MetS and CRP, MetS and sleep apnoea, and MetS and cholelithiasis were driven solely by horizontal pleiotropy (Fig. 4, Supplementary Table 10), indicating no causal relationship exists between these pairs.

### HVP model based on GWAS summary statistics

**Fig. 5:**
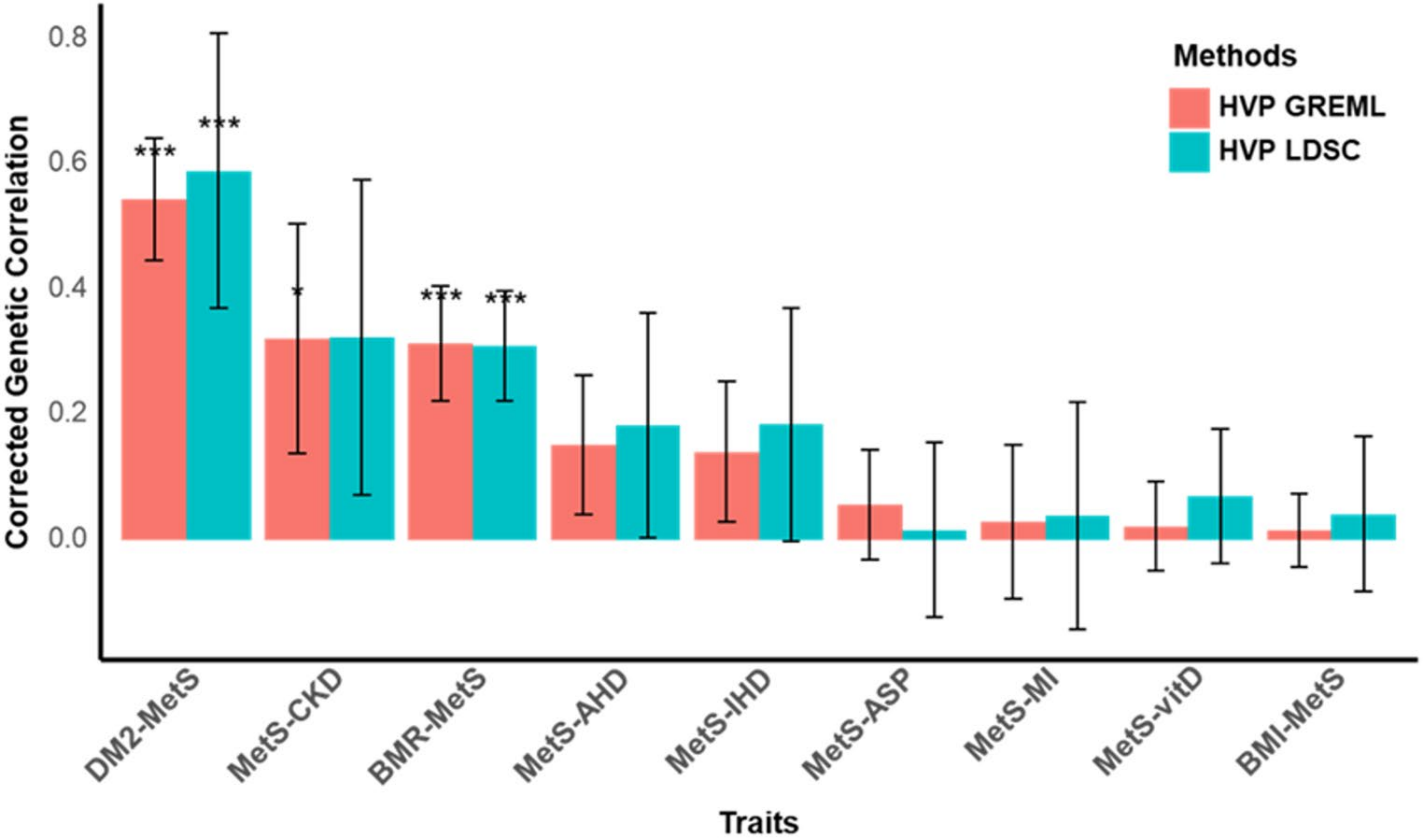
Comparison of corrected genetic correlation estimates based on LDSC and GREML. The bar plot illustrates the corrected genetic correlations between MetS and various traits, as initially estimated by LDSC (using summary statistics) and GREML (using individual level data). Estimates from the two methods are represented by distinct colours. Error bars indicate the 95% confidence intervals (CI) for the corrected genetic correlation estimates. Asterisks represent Bonferroni-adjusted significance levels (* = adjusted *p* < 0.05, ** = adjusted *p* < 0.01, *** = adjusted *p* < 0.001). Trait pairs included in the figure showed significant genetic correlations from both methods and a significant causal effect based on two-sample MR

The HVP model is compatible with GWAS summary statistics, enabling its application even when individual-level data are inaccessible. In scenarios where only summary data were available, LDSC was used to estimate genetic correlations and their associated standard errors. When applied under these conditions, the HVP model demonstrated strong concordance with results obtained from individual-level HVP analyses, showcasing its robustness and adaptability (Fig. 5). This consistency underscores the model’s utility in leveraging summary-level data for reliable genetic correlation estimation and pleiotropy dissection, broadening its applicability across diverse datasets. This functionality is also implemented in the accompanying R package, as detailed in the code availability section.

## Discussion

Genome-wide genetic correlation studies and MR analyses are essential tools in understanding the relationships between complex traits. Genetic correlation quantifies the shared genetic basis between traits, while MR leverages genetic data to estimate causal effects. Standard methods for estimating genetic correlation, such as LDSC (Bulik-Sullivan et al. 2015) and REML through approaches like GCTA (Yang et al. 2011), MTG2 (Lee and van der Werf 2016), and BOLT-REML (Loh et al. 2015), have been widely used, especially for polygenic traits. However, these methods do not always account for the distinct forms of pleiotropy—vertical and horizontal—which can obscure the true nature of genetic associations.

Vertical pleiotropy involves a causal chain where a genetic variant influences one trait, which then affects another (Jang et al. 2022). In contrast, horizontal pleiotropy occurs when a genetic variant independently affects two traits, reflecting shared biological pathways (Sivakumaran et al. 2011; Solovieff et al. 2013)). Distinguishing between these forms is critical for accurate interpretation of genetic correlations and causal relationships in disease biology.

In this study, we introduced the HVP model, an innovative approach designed to provide unbiased estimates of heritability and genetic correlation by disentangling horizontal and vertical pleiotropy. Unlike traditional methods such as GREML, the HVP model adjusts for vertical pleiotropy by incorporating causal effect estimates from MR. This allows for a clear distinction between shared genetic influences (horizontal pleiotropy) and those mediated through causal pathways (vertical pleiotropy). This distinction is critical because genetic effects arising from vertical pleiotropy may not directly impact the target trait of interest unless the intermediary trait is also addressed. Such insights have profound implications for functional studies, including gene knockdown analyses or CRISPR-based interventions, where understanding the precise causal mechanisms is essential for effective targeting and interpretation (Ford et al. 2019).

Applying the HVP model to data from the UKB, we found that horizontal pleiotropy significantly contributes to the genetic correlations between MetS and traits such as type 2 diabetes, C-reactive protein, sleep apnoea, and cholelithiasis. Previous studies reported significant genetic relationships between MetS and these traits (van Walree et al. 2022), which may primarily be driven by horizontal pleiotropy. In contrast, vertical pleiotropy was more prominent in the relationships between MetS and traits like cardiovascular diseases, serum vitamin D level, and chronic kidney disease, and that between BMI and MetS. While earlier studies reported significant genetic correlations between MetS and these traits (van Walree et al. 2022; Vattikuti et al. 2012; Chen et al. 2019), our analysis reveals that these correlations primarily arise from causal pathways linking MetS to these outcomes, rather than shared genetic effects directly influencing both MetS and these traits. These findings underscore the distinct mechanisms underlying the associations between MetS and related traits, with important implications for risk prediction and the development of targeted interventions, such as gene knockdown studies or CRISPR-based therapies.

The delta method assuming that tau is estimated without error may leads to an underestimation of the standard error of the corrected genetic correlation. However, we find that the theoretical standard error closely matches the empirical standard deviation, and importantly, the empirical coverage of the 95% confidence intervals is close to the nominal level. This suggests that the estimated standard errors are reasonably well calibrated in practice (see Supplementary Table 12). Moreover, sensitivity analyses are recommended in scenarios with small sample sizes or weak genetic associations to ensure the robustness and careful interpretation of findings.

Fitting an exposure as a covariate in standard mixed models is a common approach for estimating genetic parameters such as heritability and genetic correlation, and may partially address vertical pleiotropy. However, as shown in previous studies (Aschard et al. 2015; Wang et al. 2024), this approach can introduce collider bias. Specifically, genetic variants that affect the exposure but have no true effect on the outcome may appear spuriously associated with the outcome once the exposure is conditioned on, due to induced statistical dependencies. This results in biased SNP effect estimates, which in turn bias heritability and genetic correlation estimates. Moreover, this approach becomes less feasible and more problematic in the context of bivariate models. In contrast, the HVP model explicitly accounts for the causal path from exposure to outcome and provides a more robust framework than existing methods.

In summary, our findings validate the relevance and utility of the HVP model in unraveling the complex genetic architecture of traits like MetS, providing deeper insights into how genetic effects are shared across various health conditions such as BMI, diabetes, sleep apnoea, cholelithiasis, CKD, cardiovascular diseases, and CRP levels. The HVP model effectively separates the contributions of horizontal and vertical pleiotropy, offering a clearer understanding of the genetic mechanisms linking these traits. By integrating these insights into shared genetic pathways, our approach improves the accuracy with which we identify key genetic drivers across multiple conditions. This enhanced understanding can help inform more targeted public health strategies, enabling early interventions and personalized treatment approaches.

## Electronic supplementary material

Below is the link to the electronic supplementary material.

Supplementary Material 1

## Data Availability

No datasets were generated or analysed during the current study.

## Code availability

The proposed methods are implemented in R package, which is publicly available to download on GitHub at https://github.com/lamessad/HVP for individual-level data, https://github.com/lamessad/HVPSUM for summary data. All MR methods used are publicly available R packages with links given in the web resources section. The source code for MTG version 2.22 is publicly available in https://sites.google.com/view/s-hong-lee-homepage/mtg2. The source code of LDSC is available at https://github.com/bulik/ldsc. Web resources: TwoSampleMR, https://mrcieu.github.io/TwoSampleMR, MR-IVW, MR-Egger, MR-Weighted-Median, MR-Weighted-Mode, MR-ContMix, https://cran.r-project.org/web/packages/MendelianRandomization, MR-PRESSO, https://github.com/rondolab/MR-PRESSO, IMRP, https://github.com/XiaofengZhuCase/IMRP

GSMR, http://cnsgenomics.com/software/gsmr/, MRLOVA, https://github.com/lamessad/MRLOVA.
