# Supplementary material for: Disentangling horizontal and vertical Pleiotropy in genetic correlation estimation: introducing the HVP model
Source: Hum Genet. 2025 Sep 16;144(8):861–76. doi: 10.1007/s00439-025-02762-w (PMC12449366; doi:10.1007/s00439-025-02762-w)
Supplement: Supplementary file 1 — Supplementary Material 1 [file 439_2025_2762_MOESM1_ESM.docx]

**Disentangling Horizontal and Vertical Pleiotropy in Genetic Correlation Estimation: Introducing the HVP Model: Supplementary files**

**Supplementary Note**

We obtained SNP genotypes from the UK Biobank (UKBB) dataset. Subsequently, we performed SNP pruning for linkage disequilibrium (LD) with an LD r2 threshold of 0.25 and a window size of 250kb. Following LD pruning, we randomly selected 550 SNPs as causal variants and simulated variables **c** and **y** under the specified scenario. Our simulation involved randomly selecting 50,000 individuals for sample one and 25,000 individuals for sample two. The number of SNPs and sample size for the real genotype simulation were chosen to align with those used in the GSMR method^1^. The reduced number of SNPs in the real genotype-based simulation was selected for computational efficiency in GREML analyses with large sample sizes, and also to reflect the concept of instrumental variables in MR, which typically involve a subset of variants associated with the exposure.

The causal effect, tau is obtained from MRLOVA^2^. The estimate of the corrected correlation is derived from the analysis of 100 interactions.

**Scenario 1: Vertical pleiotropy only**

In this simulation, we exclusively modelled vertical pleiotropy (*τ* > 0) while excluding horizontal pleiotropy (*cov*(**α**,**β**)=0) and residual covariance (*cov*(**e**,**ε**)=0). Genetic effects for exposure (**c**) and outcome (**y**) variables were generated using distinct genetic variants (250 SNPs for **c** and 250 SNPS for **y**), maintaining specified variance-covariance structures, using Eq. (1).

The variance-covariance structures for the genetic (**α** and **β**) and residuals (**e** and **ε**) in Eq. (1) are as follows:

$\left[ \begin{matrix} var(\boldsymbol{\alpha}) & cov\left( \boldsymbol{\alpha},\boldsymbol{\beta} \right) \\ cov\left( \boldsymbol{\alpha},\boldsymbol{\beta} \right) & var(\boldsymbol{\beta}) \end{matrix} \right]\boldsymbol{=}\left[ \begin{matrix} 0.5 & 0 \\ 0 & 0.5 \end{matrix} \right]$

**And**

$\left[ \begin{matrix} var(\mathbf{e}) & cov\left( \mathbf{e},\boldsymbol{\varepsilon} \right) \\ cov\left( \mathbf{e},\boldsymbol{\varepsilon} \right) & var(\boldsymbol{\varepsilon}) \end{matrix} \right]\boldsymbol{=}\left[ \begin{matrix} 1-var(\boldsymbol{\alpha)}-\tau^{2}-2\tau cov\left( \boldsymbol{\alpha},\boldsymbol{\beta} \right) & 0 \\ 0 & 0.5 \end{matrix} \right]$.

where $var\left( \mathbf{e} \right)=1-var(\boldsymbol{\alpha)}-\tau^{2}-2\tau cov\left( \boldsymbol{\alpha},\boldsymbol{\beta} \right)$ , is used to maintain the phenotypic variance of y equal to 1.

This setup results in a heritability of 0.5 for both **c** and **y**. In this scenario, we systematically varied the causal effect *τ* from 0.0 to 0.4 in increments of 0.1.

**Scenario 2: Both vertical and horizontal pleiotropy**

The outcome (**y**) and exposure (**c**) genetic effects were simulated using three distinct genetic variants. These effects are decomposed into **α** = **α₁** + **α₂** and **β** = **β₁** + **β₂**. The variance-covariance structures for the genetic effects (**α₁** and **β₁**, and **α₂** and **β₂**) are specified, where **α₁** and **β₁** are generated based on the first 50 SNPs (horizontal pleiotropy), **α₂** is based on the second 250 SNPs, and **β₂** is based on the third set of 250 SNPs (vertical pleiotropy).

$\left[ \begin{matrix} var(\boldsymbol{\alpha}_{\boldsymbol{1}}) & cov\left( \boldsymbol{\alpha}_{\boldsymbol{1}},\boldsymbol{\beta}_{1} \right) \\ cov\left( \boldsymbol{\alpha}_{\boldsymbol{1}},\boldsymbol{\beta}_{1} \right) & var(\boldsymbol{\beta}_{1}) \end{matrix} \right]\boldsymbol{=}\left[ \begin{matrix} 0.35 & 0.25 \\ 0.25 & 0.26 \end{matrix} \right]$

**and**

$\left[ \begin{matrix} var(\boldsymbol{\alpha}_{\boldsymbol{2}}) & cov\left( \boldsymbol{\alpha}_{\boldsymbol{2}},\boldsymbol{\beta}_{2} \right) \\ cov\left( \boldsymbol{\alpha}_{\boldsymbol{2}},\boldsymbol{\beta}_{2} \right) & var(\boldsymbol{\beta}_{2}) \end{matrix} \right]\boldsymbol{=}\left[ \begin{matrix} 0.15 & 0 \\ 0 & 0.24 \end{matrix} \right]$

The variance-covariance structure for the residual effects (**e** and **ε**) is as follows:

$\left[ \begin{matrix} var(\mathbf{e}) & cov\left( \mathbf{e},\boldsymbol{\varepsilon} \right) \\ cov\left( \mathbf{e},\boldsymbol{\varepsilon} \right) & var(\boldsymbol{\varepsilon}) \end{matrix} \right]\boldsymbol{=}\left[ \begin{matrix} 1-h^{2}-\tau^{2}-2\tau cov\left( \boldsymbol{\alpha},\boldsymbol{\beta} \right) & 0 \\ 0 & 0.5 \end{matrix} \right]$.

where $\mathrm{var}\left( \mathbf{e} \right)=1-h^{2}-\tau^{2}-2\tau cov\left( \boldsymbol{\alpha},\boldsymbol{\beta} \right)$ is used to maintain the phenotypic variance of **y** equal to 1.

This setup results in a heritability of 0.5 for both **c** and **y**, and genetic correlation attributed to horizontal pleiotropy is 0.5 In this scenario, we systematically varied the causal effect *τ* from 0.0 to 0.4 in increments of 0.1.

**Scenario 3: Vertical and horizontal pleiotropy plus residual covariance**

In the third scenario, we introduced a more complex genetic setting involving both vertical and horizontal pleiotropy (*τ* > 0 and *cov*(**α**,**β**) > 0), along with the inclusion of residual covariance (*cov*(**e**,**ε**)>0). To distinguish between vertical and horizontal pleiotropy, we utilized three distinct sets of SNPs. The genetic effects can be decomposed as follows: **α** = **α₁** + **α₂** and **β** = **β₁** + **β₂**. The variance-covariance structures for the genetic effects (**α₁** and **β₁**, and **α₂** and **β₂**) are specified, where **α₁** and **β₁** are generated based on the first 50 SNPs (horizontal pleiotropy), **α₂** is based on the second 250 SNPs, and **β₂** is based on the third set of 250 SNPs (vertical pleiotropy).

$\left[ \begin{matrix} var(\boldsymbol{\alpha}_{\boldsymbol{1}}) & cov\left( \boldsymbol{\alpha}_{\boldsymbol{1}},\boldsymbol{\beta}_{1} \right) \\ cov\left( \boldsymbol{\alpha}_{\boldsymbol{1}},\boldsymbol{\beta}_{1} \right) & var(\boldsymbol{\beta}_{1}) \end{matrix} \right]\boldsymbol{=}\left[ \begin{matrix} 0.35 & 0.25 \\ 0.25 & 0.26 \end{matrix} \right]$

**and**

$\left[ \begin{matrix} var(\boldsymbol{\alpha}_{\boldsymbol{2}}) & cov\left( \boldsymbol{\alpha}_{\boldsymbol{2}},\boldsymbol{\beta}_{2} \right) \\ cov\left( \boldsymbol{\alpha}_{\boldsymbol{2}},\boldsymbol{\beta}_{2} \right) & var(\boldsymbol{\beta}_{2}) \end{matrix} \right]\boldsymbol{=}\left[ \begin{matrix} 0.15 & 0 \\ 0 & 0.24 \end{matrix} \right]$

The variance-covariance structure for the residual effects (**e** and **ε**) is as follows:

$\left[ \begin{matrix} var(\mathbf{e}) & cov\left( \mathbf{e},\boldsymbol{\varepsilon} \right) \\ cov\left( \mathbf{e},\boldsymbol{\varepsilon} \right) & var(\boldsymbol{\varepsilon}) \end{matrix} \right]\boldsymbol{=}\left[ \begin{matrix} 1-h^{2}-\tau^{2}-2\tau cov\left( \boldsymbol{\alpha},\boldsymbol{\beta} \right)\mathbf{-}2\tau cov\left( e,\varepsilon\right) & 0.2 \\ 0.2 & 0.5 \end{matrix} \right]$.

where $\mathrm{var}\left( \mathbf{e} \right)=1-h^{2}-\tau^{2}-2\tau cov\left( \boldsymbol{\alpha},\boldsymbol{\beta} \right)\mathbf{-}2\tau cov\left( e,\varepsilon\right)$

This setup results in a heritability of 0.5 for both **c** and **y**, with a genetic correlation due to horizontal pleiotropy set at 0.5. In this scenario, we systematically varied the causal effect *τ* from 0.0 to 0.4 in increments of 0.1.

**Scenario 4: Complete mediation of genetic effect of trait 1(y) –** not simulated.

**Scenario 5: There are no genetic variants that influence the outcome independently of the exposure.**

The fifth scenario is a special form of scenario 2 where all causal SNPs of **y** are in horizontal pleiotropy with **c** (*var*(**α₂**) = 0). The variance-covariance structures for the genetic effects (**α₁** and **β₁**, and **α₂** and **β₂**) are as follows:

$\left[ \begin{matrix} var(\boldsymbol{\alpha}_{\boldsymbol{1}}) & cov\left( \boldsymbol{\alpha}_{\boldsymbol{1}},\boldsymbol{\beta}_{1} \right) \\ cov\left( \boldsymbol{\alpha}_{\boldsymbol{1}},\boldsymbol{\beta}_{1} \right) & var(\boldsymbol{\beta}_{1}) \end{matrix} \right]\boldsymbol{=}\left[ \begin{matrix} 0.5 & 0.25 \\ 0.25 & 0.26 \end{matrix} \right]$

**and**

$\left[ \begin{matrix} var(\boldsymbol{\alpha}_{\boldsymbol{2}}) & cov\left( \boldsymbol{\alpha}_{\boldsymbol{2}},\boldsymbol{\beta}_{2} \right) \\ cov\left( \boldsymbol{\alpha}_{\boldsymbol{2}},\boldsymbol{\beta}_{2} \right) & var(\boldsymbol{\beta}_{2}) \end{matrix} \right]\boldsymbol{=}\left[ \begin{matrix} 0 & 0 \\ 0 & 0.24 \end{matrix} \right]$

The variance-covariance structure for the residual effects (**e** and **ε**) is as follows:

$\left[ \begin{matrix} var(\mathbf{e}) & cov\left( \mathbf{e},\boldsymbol{\varepsilon} \right) \\ cov\left( \mathbf{e},\boldsymbol{\varepsilon} \right) & var(\boldsymbol{\varepsilon}) \end{matrix} \right]\boldsymbol{=}\left[ \begin{matrix} 1-var(\boldsymbol{\alpha}_{\boldsymbol{1}}\boldsymbol{)}-\tau^{2}-2\tau cov\left( \boldsymbol{\alpha}_{\mathbf{1}},\boldsymbol{\beta}_{\mathbf{1}}\mathbf{+}\boldsymbol{\beta}_{2} \right) & 0 \\ 0 & 0.5 \end{matrix} \right]$.

where $var\left( \mathbf{e} \right)=1-var(\boldsymbol{\alpha}_{\boldsymbol{1}}\boldsymbol{)}-\tau^{2}-2\tau cov\left( \boldsymbol{\alpha}_{\mathbf{1}},\boldsymbol{\beta}_{\mathbf{1}}\mathbf{+}\boldsymbol{\beta}_{2} \right)$ is used to maintain the phenotypic variance of **y** equal to 1.

This setup results in a heritability of 0.5 for both c and y, and genetic correlation attributed to horizontal pleiotropy is 0.5. In this scenario, we systematically varied the causal effect τ from 0 to 0.4 in increments of 0.1.

Supplementary Table 1: Comorbidities, abbreviations, and ICD definitions of the 12 complex traits.

|  | **Comorbidity** | **Abbreviation** | **ICD-9 definitions** | **ICD-10 definitions** |
| --- | --- | --- | --- | --- |
| 1 | Ischemic heart diseases | IHD | 410.x, 411.x, 412.x, 413.x, 414.x | I20.x, I21.x, I22.x, I23.x, I24.x, I25.x |
| 2 | Atherosclerotic heart disease | AHD | 414.0 | I25.1 |
| 3 | Atrial Fibrillation/Flutter | AFib | 427.3 | I48.x |
| 4 | Stroke |  | 430.x, 431.x, 433.x, 434.x, 435.x, | I60.x, I61.x, I63.x, I64.x |
| 5 | Type 2 Diabetes | DM2 | 250.00, 250.09, 250.x2 | E11.x |
| 6 | Chronic obstructive pulmonary diseases | COPD | 492.x, 496.x | J42-J44.x |
| 7 | Chronic kidney disease | CKD | 585 | N18.x |
| 8 | Sleep apnoea | SA | NA | G47.3 |
| 9 | Cholelithiasis |  | 574.x | K80.x |
| 10 | Anxiety disorder | AD | 300.0, 300.2, 300.3, 309.8 | F40.x, F41.X, F42.X, |
| 11 | Major depressive disorder | MDD | 296.2x, 296.3x, 311.xx | F32.x, F33.x |
| 12 | Myocardial Infarction | MI | 410,411,412 | I21,x I22.x, I23.x, I24,.1, I25.2 |

Supplementary Table 2: Phenotypes, abbreviations, UKB field ID of the 11 quantitative complex traits.

|  | **Trait** | **Abbreviation** | Field ID |
| --- | --- | --- | --- |
| 1 | FEV1/ FVC ratio Z-score | FEV1/ FVC | 20258 |
| 2 | C-reactive protein | CRP | 30710 |
| 3 | Alanine aminotransferase | ALT | 30620 |
| 4 | Aspartate aminotransferase | AST | 30650 |
| 5 | Alkaline phosphatase | ALP | 30610 |
| 6 | Gamma glutamyl transferase | GGT | 30730 |
| 7 | Serum Vitamin D level | Vitamin D | 30890 |
| 8 | Basal metabolic rate | BMR | 23105 |
| 9 | Body mass index | BMI | 21001 |
| 10 | Insulin-like growth factor 1 | IGF-1 | 30770 |
| 11 | Neuroticism score |  | 20127 |

Supplementary Table 3: Genetic correlation between traits **y** and **c** simulated under scenario 2 of supplementary note.

|  | Binary outcome, Quantitative exposure | | Quantitative outcome, Binary exposure | | Binary outcome, Binary exposure | |
| --- | --- | --- | --- | --- | --- | --- |
| tau | GREML  r_G_(se) | HVP  r_G_(se) | GREML  r_G_(se) | HVP  r_G_(se) | GREML  rG(se) | HVP  r_G_(se) |
| 0 | 0.49(0.002) | 0.49(0.004) | 0.49(0.005) | 0.49(0.005) | 0.49(0.006) | 0.49(0.006) |
| 0.1 | 0.56(0.003) | 0.49(0.004) | 0.57(0.003) | 0.50(0.003) | 0.57(0.003) | 0.50(0.004) |
| 0.2 | 0.62(0.003) | 0.49(0.003) | 0.63(0.002) | 0.50(0.003) | 0.62(0.003) | 0.50(0.004) |
| 0.3 | 0.66(0.002) | 0.49(0.004) | 0.67(0.002) | 0.50(0.003) | 0.67(0.002) | 0.51(0.004) |
| 0.4 | 0.70(0.002) | 0.48(0.005) | 0.69(0.002) | 0.50(0.003) | 0.70(0.002) | 0.49(0.004) |

tau represents the fixed causal effect between the two traits under the liability scale, varying from 0 to 0.4. rG is genetic correlation se standard error.

Supplementary Table 4: Heritability and genetic correlation between traits *y* and *c* under negative τ based on simulated genotype (Scenarios 2 and 3; 100 iterations)

| τ | Parameter  Est(se) | scenario 2 | | scenario 3 | |
| --- | --- | --- | --- | --- | --- |
|  |  | GREML | HVP | GREML | HVP |
| -0.1 | Heritability | 0.46(0.001) | 0.5(0.002) | 0.46(0.001) | 0.5(0.002) |
|  | Genetic correlation | 0.42(0.003) | 0.5(0.002) | 0.42(0.002) | 0.5(0.002) |
| -0.2 | Heritability | 0.42(0.001) | 0.5(0.003) | 0.42(0.001) | 0.5(0.001) |
|  | Genetic correlation | 0.33(0.002) | 0.5(0.002) | 0.33(0.002) | 0.5(0.001) |
| -0.3 | Heritability | 0.39(0.001) | 0.5(0.002) | 0.39(0.001) | 0.5(0.002) |
|  | Genetic correlation | 0.23(0.003) | 0.5(0.002) | 0.23(0.003) | 0.5(0.002) |
| -0.4 | Heritability | 0.38(0.001) | 0.5(0.002) | 0.38(0.001) | 0.5(0.002) |
|  | Genetic correlation | 0.11(0.003) | 0.5(0.002) | 0.12(0.002) | 0.5(0.002) |

Supplementary Table 5: Heritability and genetic correlation between traits *y* and *c* under negative τ based on real genotype data (Scenarios 2* and 3*; 50 iterations)

| τ | Parameter  Est(se) | scenario 2 | | scenario 3 | |
| --- | --- | --- | --- | --- | --- |
|  |  | GREML | HVP | GREML | HVP |
| -0.1 | Heritability | 0.45(0.001) | 0.49(0.001) | 0.46(0.001) | 0.49(0.002) |
|  | Genetic correlation | 0.42(0.001) | 0.50(0.004) | 0.41(0.004) | 0.49(0.004) |
| -0.2 | Heritability | 0.41(0.001) | 0.49(0.001) | 0.42(0.001) | 0.49(0.001) |
|  | Genetic correlation | 0.32(0.001) | 0.50(0.003) | 0.32(0.005) | 0.49(0.004) |
| -0.3 | Heritability | 0.39(0.001) | 0.49(0.001) | 0.39(0.001) | 0.49(0.001) |
|  | Genetic correlation | 0.21(0.005) | 0.49(0.005) | 0.22(0.004) | 0.49(0.004) |
| -0.4 | Heritability | 0.38(0.001) | 0.49(0.001) | 0.38(0.001) | 0.49(0.002) |
|  | Genetic correlation | 0.11(0.006) | 0.48(0.005) | 0.10(0.005) | 0.48(0.005) |

***Scenario 2:** Traits **y** and **c** are simulated under both horizontal and vertical pleiotropy without residual covariance. The variance-covariance structures for genetic effects $\left[ \begin{matrix} var(\boldsymbol{\alpha}_{\boldsymbol{1}}) & cov\left( \boldsymbol{\alpha}_{\boldsymbol{1}},\boldsymbol{\beta}_{1} \right) \\ cov\left( \boldsymbol{\alpha}_{\boldsymbol{1}},\boldsymbol{\beta}_{1} \right) & var(\boldsymbol{\beta}_{1}) \end{matrix} \right]\boldsymbol{=}\left[ \begin{matrix} 0.35 & 0.25 \\ 0.25 & 0.26 \end{matrix} \right]$ and $\left[ \begin{matrix} var(\boldsymbol{\alpha}_{\boldsymbol{2}}) & cov\left( \boldsymbol{\alpha}_{\boldsymbol{2}},\boldsymbol{\beta}_{2} \right) \\ cov\left( \boldsymbol{\alpha}_{\boldsymbol{2}},\boldsymbol{\beta}_{2} \right) & var(\boldsymbol{\beta}_{2}) \end{matrix} \right]\boldsymbol{=}\left[ \begin{matrix} 0.15 & 0 \\ 0 & 0.24 \end{matrix} \right]$. Residual effects (**e** and **ε**) are characterized by $\left[ \begin{matrix} var\left( \mathbf{e} \right) & 0 \\ 0 & 0.5 \end{matrix} \right]$, maintaining **y**'s phenotypic variance at 1.

***Scenario 3:** Traits **y** and **c** are simulated under both horizontal and vertical pleiotropy, with residual covariance. The variance-covariance structures for genetic effects are $\left[ \begin{matrix} var(\boldsymbol{\alpha}_{\boldsymbol{1}}) & cov\left( \boldsymbol{\alpha}_{\boldsymbol{1}},\boldsymbol{\beta}_{1} \right) \\ cov\left( \boldsymbol{\alpha}_{\boldsymbol{1}},\boldsymbol{\beta}_{1} \right) & var(\boldsymbol{\beta}_{1}) \end{matrix} \right]\boldsymbol{=}\left[ \begin{matrix} 0.35 & 0.25 \\ 0.25 & 0.26 \end{matrix} \right]$ and $\left[ \begin{matrix} var(\boldsymbol{\alpha}_{\boldsymbol{2}}) & cov\left( \boldsymbol{\alpha}_{\boldsymbol{2}},\boldsymbol{\beta}_{2} \right) \\ cov\left( \boldsymbol{\alpha}_{\boldsymbol{2}},\boldsymbol{\beta}_{2} \right) & var(\boldsymbol{\beta}_{2}) \end{matrix} \right]\boldsymbol{=}\left[ \begin{matrix} 0.15 & 0 \\ 0 & 0.24 \end{matrix} \right]$. Residual effects (**e** and **ε**) are characterized by $\left[ \begin{matrix} var\left( \mathbf{e} \right) & 0.1 \\ 0.1 & 0.5 \end{matrix} \right]$, maintaining **y**'s phenotypic variance at 1.

Supplementary Table 6: Sociodemographic characteristics of study participants (n=82,955).

| **Characteristics** | **N (%)** |
| --- | --- |
| **Sex** |  |
| Female | 43,501 (52.44) |
| Male | 39,454 (47.56) |
| **Age at baseline (years)** |  |
| 39-49 | 19,516 (23.53) |
| 50-59 | 28,391 (34.23) |
| 60-73 | 35,048 (42.25) |
| **Educational status** |  |
| None | 10,804 (13.02) |
| CSEs/ GCSEs/ 0 levels | 22,694 (27.36) |
| A level | 10,243 (12.35) |
| Other professional qualifications e.g. nursing, teaching | 4,242 (5.11) |
| NVQ/ HND/ HNC/ degree | 34,638 (41.76) |
| Missing | 334 (0.4) |
| **Body mass index (kg/m^2^)** |  |
| Underweight (<18.5) | 354 (0.43) |
| Normal (18.5 to <25) | 27,376 (33.00) |
| Overweight (25 to <30) | 35,829 (43.19) |
| Obese (>=30) | 19,225 (23.18) |
| Missing | 171 (0.21) |
| **Metabolic Syndrome** |  |
| Yes | 17,335 (20.90) |
| No | 65,620 (79.10) |

CSE = Certificate of Secondary Education; GCSE = General Certificate of Secondary Education; NVQ =National Vocational Qualification; HNC=Higher National Certificate; HND =Higher National Diploma

Supplementary Table 7: Heritability of MetS and related comorbidities and complex

| **Trait** | **Heritability h^2^(se) in %** | | **p-value** |
| --- | --- | --- | --- |
|  | **Observed scale** | **Liability scale** |  |
| Basal metabolic rate | 29.18(0.56) |  | 0.00E+00 |
| Body mass index | 22.46(0.54) |  | 0.00E+00 |
| Insulin-like growth factor 1 | 23.13(0.56) |  | 0.00E+00 |
| Alkaline phosphatase | 17.92(0.54) |  | 1.76E-241 |
| FEV1/FVC ratio Z-score | 18.78(0.60) |  | 4.67E-215 |
| Serum vitamin D level | 10.08(0.50) |  | 2.20E-90 |
| Neuroticism score | 10.46(0.53) |  | 1.06E-86 |
| Gamma glutamyl transferase | 8.92(0.48) |  | 4.38E-77 |
| Alanine aminotransferase | 8.34(0.47) |  | 1.89E-70 |
| Aspartate aminotransferase | 6.32(0.46) |  | 5.92E-43 |
| Type II diabetes | 5.94(0.44) | 20.72(1.53) | 1.56E-41 |
| C-reactive protein | 5.39(0.45) |  | 4.65E-33 |
| Atrial Fibrillation/Flutter | 4.3(0.42) | 14.4(1.41) | 1.34E-24 |
| Ischemic heart diseases | 3.95(0.41) | 10.83(1.13) | 5.74E-22 |
| Atherosclerotic heart disease | 4.02(0.42) | 14.59(1.53) | 1.05E-21 |
| Myocardial infarction | 3.01(0.40) | 13.69(1.82) | 5.27E-14 |
| Cholelithiasis | 1.95(0.39) | 8.17(1.63) | 5.73E-07 |
| Chronic kidney disease | 1.87(0.39) | 9.147(1.91) | 1.63E-06 |
| Chronic obstructive pulmonary diseases | 1.39(0.38) | 7.15(1.96) | 2.54E-04 |
| Anxiety disorder | 1.27(0.38) | 5.68(1.70) | 8.31E-04 |
| Major depressive disorder | 1.22(0.38) | 3.74(1.17) | 1.32E-03 |
| Sleep apnoea | 1.08(0.38) | 9.09(3.20) | 4.48E-03 |
| Stroke | 0.77(0.38) | 5.32(2.62) | 4.27E-02 |

traits(n=82,955)

*Adjusted for age, sex, education, TDI, and population structure (first 10 PCA)

Supplementary Table 8: Frequency comparison of comorbidities in participants with and without MetS (n=82,955).

|  | **Frequency (%)** | | | **OR** | **Adjusted OR*** | **p-value** |
| --- | --- | --- | --- | --- | --- | --- |
|  | **Total** | **MetS +** | **MetS -** |  |  |  |
| **Atherosclerotic heart disease** | | | | | | |
| Yes | 5755(6.94) | 2026(11.69) | 3729(5.68) | 2.20 (0.064) | 1.53(0.052) | < 2.2E-16 |
| No |  | 15309(88.31) | 61891(94.32) |  |  |  |
| **Atrial Fibrillation/Flutter** | | | | | | |
| Yes | 6579(7.93) | 2061(11.89) | 4518(6.89) | 1.83(0.051) | 1.08(0.037) | 1.97E-02 |
| No |  | 15274(88.11) | 61102(93.11) |  |  |  |
| **Anxiety disorder** | | | | | | |
| Yes | 4141(4.99) | 1118(6.44) | 3023(4.61) | 1.43(0.052) | 1.24(0.053) | 4.43E-07 |
| No |  | 16217(93.55) | 62597(95.39) |  |  |  |
| **Cholelithiasis** | | | | | | |
| Yes | 4578(5.52) | 1560(9.00) | 3018(4.60) | 2.05(0.067) | 1.31(0.050) | 4.20E-12 |
| No |  | 15775(91.00) | 62602(95.40) |  |  |  |
| **Chronic kidney disease** | | | | | | |
| Yes | 3606(4.35) | 1496(8.63) | 2110(3.22) | 2.84(0.099) | 1.74(0.073) | < 2.2e-16 |
| No |  | 15839(91.37) | 63510(96.78) |  |  |  |
| **Chronic obstructive pulmonary diseases** | | | | | | |
| Yes | 3340(4.03) | 1098(6.33) | 2242(3.42) | 1.91(0.072) | 1.34(0.061) | 1.77E-10 |
| No |  | 16237(93.67) | 63378(96.58) |  |  |  |
| **Major depressive disorder** | | | | | | |
| Yes | 4910(5.92) | 1480(8.54) | 3430(5.23) | 1.69(0.055) | 1.30(0.051) | 1.08E-11 |
| No |  | 15855(91.46) | 62190(94.77) |  |  |  |
| **Diabetes mellites type 2** | | | | | | |
| Yes | 6150(7.41) | 3953(22.80) | 2197(3.35) | 8.53(0.241) | 4.31(0.141) | < 2.2e-16 |
| No |  | 13382(77.20) | 63423(96.65) |  |  |  |
| **Ischemic heart diseases** | | | | | | |
| Yes | 9284(11.19) | 3259(18.80) | 6025(9.18) | 2.29(0.054) | 1.54(0.043) | < 2.2e-16 |
| No |  | 14076(81.20) | 59595(90.82) |  |  |  |
| **Myocardial infarction** | | | | | | |
| Yes | 4029(4.86) | 1482(8.55) | 2547(3.88) | 2.32(0.078) | 1.554(0.061) | < 2.2e-16 |
| No |  | 15853(91.45) | 63073(96.12) |  |  |  |
| **Sleep apnoea** | | | | | | |
| Yes | 1643(1.98) | 804(4.64) | 839(1.28) | 3.76(0.188) | 1.27(0.079) | 1.50E-04 |
| No |  | 16531(95.36) | 64781(98.72) |  |  |  |
| **Stroke** | | | | | | |
| Yes | 2172(2.62) | 635(3.66) | 1537(2.34) | 1.56(0.076) | 1.15(0.064) | 1.53E-02 |
| No |  | 16700(96.34) | 64083(97.66) |  |  |  |

*Adjusted for age, sex, education, TDI, and BMI. In the analysis conducted within the UK Biobank (UKBB), we investigated the prevalence of 12 ICD-10 codes, previously linked to MetS, in two distinct groups: individuals with MetS (n=17,335) and those without MetS (n=65,620). Notably, all the comorbidities exhibited a higher occurrence among those with MetS compared to the without MetS group. The frequencies of ICD 10 codes were compared between the participants with and without MetS, and of the ICD-10 comorbidities considered, all showed statistically significant chi-square tests after multiple test correction. Subsequently, the association of stroke and atrial fibrillation with MetS disappeared after adjusting for age, sex, education, BMI, and TDI, while the other comorbidities remain significant after adjusting for the covariates and multiple test correction.

Supplementary Table 9: Mean comparison of quantitative traits in participants with and without MetS(n=82,955).

|  |  | **Mean(±SD)** | | **Mean Difference* (SE)** | **p-value** |
| --- | --- | --- | --- | --- | --- |
|  | **Total** | **MetS +** | **MetS -** |  |  |
| **Alkaline phosphatase** | | | | | |
|  | 83.05(25.73) | 88.46(0.214) | 81.56(28.1) | 2.86(0.246) | < 2.00E-16 |
| **Alanine aminotransferase** | | | | | |
|  | 23.54(13.9) | 29.27(16.9) | 21.96(12.5) | 4.13(0.128) | < 2.00E-16 |
| **Aspartate aminotransferase** | | | | | |
|  | 26.07(9.57) | 28.038(11.7) | 25.53(8.81) | 1.41(0.092) | < 2.00E-16 |
| **Basal metabolic rate** | | | | | |
|  | 6686.83(1368.9) | 7366.24(1495.) | 6508.29(1275) | 106.21(5.587) | < 2.00E-16 |
| **C-reactive protein** | | | | | |
|  | 2.43(4.03) | 3.53(4.61) | 2.12(3.80) | 0.31(0.038) | 3.40E-16 |
| **FEV1/ FVC ratio Z-score** | | | | | |
|  | 0.41(0.88) | 0.323(0.884) | 0.44(0.871) | -0.004(0.009) | 6.77E-01 |
| **Gamma** **glutamyl transferase** | | | | | |
|  | 36.45(37.52) | 47.55(46.7) | 33.38(33.9) | 7.43(0.357) | < 2.00E-16 |
| **Insulin-like growth factor 1** | | | | | |
|  | 21.67(5.65) | 20.58(5.86) | 21.98(5.55) | -0.39(0.053) | 3.37E-13 |
| **Neuroticism score** | | | | | |
|  | 4.03(3.22) | 4.10(3.26) | 4.01(3.22) | 0.16(0.033) | 3.54E-03 |
| **Serum vitamin D level** | | | | | |
|  | 49.83(20.76) | 44.65(19.1) | 51.25(21.0) | -3.88(0.204) | < 2.00E-16 |

*Adjusted for age, sex, education, TDI, and BMI, MD is mean difference between with and without MetS. In the two groups (with and without MetS) we compared the mean of 10 quantitative complex traits collected at baseline and found significant mean difference after multiple test correction except for FEV1FVC ratio z-score. The significance of mean differences for these traits persisted even after adjusting for age, sex, education, TDI and BMI.

Supplementary table 10: Genetic correlation of MetS and related complex traits using GREML, LDSC, and HVP.

| **Traits** | | **GREML** | | | **LDSC** | | | **MR** | | | **HVP-GREML** | | | **HVP-LDSC** | | |
| --- | --- | --- | --- | --- | --- | --- | --- | --- | --- | --- | --- | --- | --- | --- | --- | --- |
| **Exposure** | **Outcome** | **rg** | **se** | **p-value** |  |  |  | tau | se | **p-value** | **rg** | **se** | **p-value** | **rg** | **se** | **p-value** |
| MetS | CKD | 0.54 | 0.084 | 1.12E-10 | 0.53 | 0.118 | 6.80E-06 | 0.12 | 0.032 | 4.16E-04 | 0.32 | 0.094 | 5.99E-04 | 0.32 | 0.128 | 1.16E-02 |
| MetS | SA | 0.47 | 0.116 | 4.67E-05 | 0.53 | 0.231 | 2.30E-02 | 0.00 | 0.032 | 9.30E-01 |  |  |  |  |  |  |
| MetS | ALT | 0.42 | 0.036 | 5.53E-33 | 0.46 | 0.060 | 2.03E-14 | -0.03 | 0.039 | 4.79E-01 |  |  |  |  |  |  |
| MetS | IHD | 0.37 | 0.052 | 1.36E-12 | 0.43 | 0.086 | 5.73E-07 | 0.16 | 0.033 | 4.22E-05 | 0.14 | 0.057 | 1.14E-02 | 0.182 | 0.095 | 5.306E-02 |
| MetS | AHD | 0.37 | 0.052 | 2.42E-12 | 0.37 | 0.085 | 1.12E-05 | 0.15 | 0.037 | 4.58E-04 | 0.15 | 0.069 | 7.53E-03 | 0.184 | 0.092 | 4.64E-02 |
| MetS | Cholelithiasis | 0.34 | 0.076 | 5.71E-06 | 0.19 | 0.092 | 4.08E-02 | 0.06 | 0.033 | 8.09E-02 |  |  |  |  |  |  |
| MetS | MI | 0.30 | 0.060 | 5.35E-07 | 0.29 | 0.091 | 1.55E-03 | 0.16 | 0.028 | 3.25E-06 | 0.03 | 0.063 | 6.51E-01 | 0.04 | 0.093 | 6.79E-01 |
| MetS | MDD | 0.24 | 0.095 | 1.13E-02 |  |  |  | -0.02 | 0.032 | 6.29E-01 |  |  |  |  |  |  |
| MetS | GGT | 0.23 | 0.037 | 3.43E-10 | 0.27 | 0.070 | 1.60E-04 | 0.03 | 0.038 | 3.99E-01 |  |  |  |  |  |  |
| MetS | ASP | 0.22 | 0.043 | 1.94E-07 | 0.19 | 0.072 | 8.39E-03 | 0.14 | 0.039 | 3.21E-04 | 0.06 | 0.0449 | 2.10E-01 | 0.02 | 0.071 | 8.26E-01 |
| MetS | AFib | 0.22 | 0.051 | 1.08E-05 | 0.19 | 0.092 | 4.08E-02 | 0.02 | 0.034 | 5.57E-01 |  |  |  |  |  |  |
| MetS | ACD | 0.22 | 0.053 | 4.67E-05 | 0.16 | 0.099 | 1.07E-01 | 0.02 | 0.032 | 5.08E-01 |  |  |  |  |  |  |
| MetS | COPD | 0.15 | 0.087 | 7.94E-02 |  |  |  | 0.04 | 0.032 | 2.89E-01 |  |  |  |  |  |  |
| MetS | ALP | 0.10 | 0.029 | 4.21E-04 | 0.06 | 0.056 | 2.52E-01 | 0.05 | 0.038 | 2.28E-01 |  |  |  |  |  |  |
| MetS | Stroke | 0.04 | 0.114 | 7.30E-01 |  |  |  | -0.05 | 0.034 | 1.62E-01 |  |  |  |  |  |  |
| MetS | Anxiety | 0.01 | 0.089 | 8.75E-01 |  |  |  | 0.01 | 0.031 | 7.30E-01 |  |  |  |  |  |  |
| MetS | Neuroticism | 0.01 | 0.037 | 8.56E-01 |  |  |  | -0.05 | 0.035 | 1.57E-01 |  |  |  |  |  |  |
| MetS | IGF-1 | -0.06 | 0.026 | 3.38E-02 |  |  |  | 0.03 | 0.043 | 5.56E-01 |  |  |  |  |  |  |
| MetS | FEV1FVC | -0.10 | 0.029 | 9.46E-04 | -0.16 | 0.046 | 4.22E-04 | -0.03 | 0.034 | 4.42E-01 |  |  |  |  |  |  |
| MetS | vitD | -0.12 | 0.036 | 7.55E-04 | -0.05 | 0.059 | 3.74E-01 | -0.15 | 0.036 | 4.42E-04 | 0.02 | 0.036 | 5.55E-01 | 0.07 | 0.055 | 2.04E-1 |
| DM2 | MetS | 0.69 | 0.037 | 2.17E-78 | 0.73 | 0.080 | 6.65E-20 | 0.29 | 0.043 | 2.39E-06 | 0.54 | 0.050 | 1.53E-27 | 0.59 | 0.112 | 1.45E-07 |
| CRP | MetS | 0.37 | 0.045 | 1.95E-16 | 0.39 | 0.106 | 2.71E-04 | 0.00 | 0.030 | 9.99E-01 |  |  |  |  |  |  |
| BMR | MetS | 0.50 | 0.020 | 1.88E-144 | 0.51 | 0.041 | 5.81E-35 | 0.12 | 0.017 | 2.38E-11 | 0.31 | 0.023 | 2.44E-42 | 0.31 | 0.0.47 | 4.62E-12 |
| BMI | MetS | 0.65 | 0.019 | 4.44E-254 | 0.69 | 0.040 | 2.12E-65 | 0.40 | 0.023 | 1.38E-29 | 0.02 | 0.030 | 6.08E-01 | 0.04 | 0.063 | 5.15E-01 |

The cause effects were estimated by MRLOVAR and corrected genetic correlation of MetS and related complex traits using HVP model.

Supplementary Table 11: The number of instrumental variants, along with their F-statistics and coefficients of determination.

| **Trait** | **Number of Genetic Instruments** | **R²** | **F-statistics** |
| --- | --- | --- | --- |
| MetS | 13 | 1.20% | 74 |
| BMR | 182 | 5.40% | 64 |
| BMI | 116 | 3.00% | 55.3 |
| CRP | 19 | 1.60% | 178.5 |
| DM2 | 25 | 0.80% | 63.4 |
| IHD | 18 | 0.40% | 48.9 |
| MI | 7 | 0.20% | 48.9 |
| AHD | 25 | 0.60% | 60.2 |
| Vitamin D | 23 | 1.50% | 159.1 |

AHD atherosclerotic heart disease, IHD ischemic heart disease, MI myocardial infarction, CKD chronic kidney disease, DM2 type II diabetes, BMI body mass index, BMR basal metabolic rate, CRP C-reactive protein.

Table 1**2:** Comparison of empirical and theoretical standard errors based on simulated genotype and real genotype data

|  | Simulated genotype | | | UKB genotype | | |
| --- | --- | --- | --- | --- | --- | --- |
| τ simulated | Empirical standard error | Theoretical standard error | 95% CI Coverage (%) | Empirical standard error | Theoretical standard error | 95% CI Coverage (%) |
| 0.1 | 0.021 | 0.022 | 98 | 0.036 | 0.032 | 90 |
| 0.2 | 0.022 | 0.021 | 94 | 0.031 | 0.032 | 98 |
| 0.3 | 0.021 | 0.021 | 95 | 0.037 | 0.032 | 92 |
| 0.4 | 0.018 | 0.020 | 97 | 0.026 | 0.032 | 100 |

For the simulation based on simulated genotype, 𝜏 was estimated using GREML applied to the second set of SNPs over 100 iterations. For the real genotype-based simulation, 𝜏 was estimated using MR methods over 50 iterations.


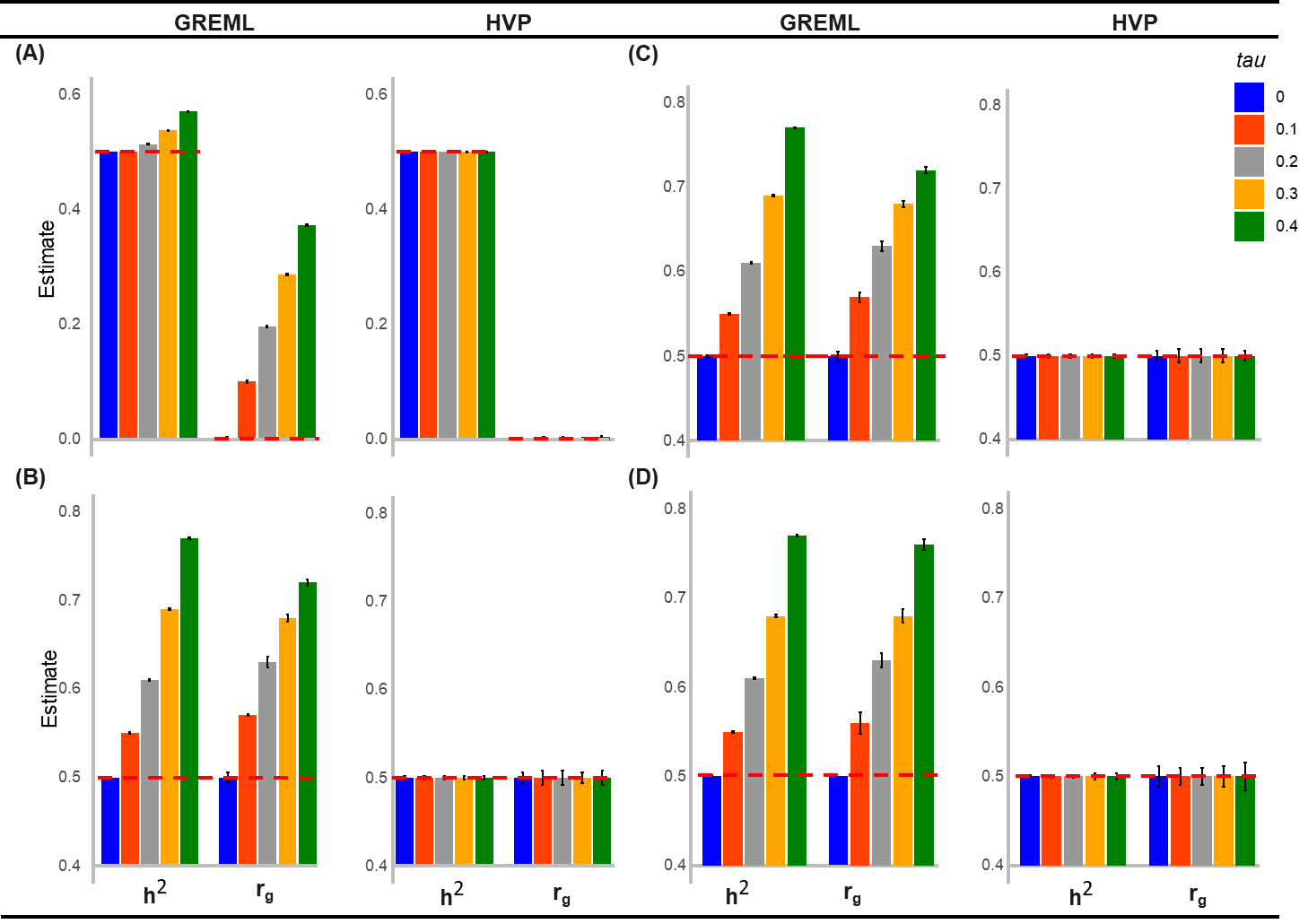


Supplementary Figure 1: Estimated Heritability(h^2^) and Genetic Correlation(r_g_)

This figure presents the outcomes of a simulation study examining the heritability of trait **y** and the genetic correlation between traits **y** and **c** across four distinct scenarios (supplementary note). Each scenario explores different levels of genetic covariance and pleiotropy between the traits. In Scenario 1 (panel A), which investigates the absence of genetic covariance, the left plot illustrates the biased heritability of trait **y** and genetic covariance due to vertical pleiotropy. Conversely, the right plot demonstrates how τ estimation facilitates the disentanglement of horizontal pleiotropy from the vertical in the HVP model. In Scenario 2 (panel B), focusing on the presence of genetic covariance, the left plot reveals the biased heritability of trait y and genetic covariance, while the right plot showcases how τ estimation guides the dismantling of horizontal pleiotropy from the vertical in the HVP model. Scenario 3 (panel C) delves into the presence of both genetic and residual covariances. The left plot displays the biased heritability of trait **y** and genetic covariance due to vertical pleiotropy, while the right plot exhibits how tau estimation aids in dismantling horizontal pleiotropy from the vertical in the HVP model. Lastly, Scenario 5 (panel D) investigates in the presence of genetic covariance, where the left plot depicts the biased heritability of trait **y** and genetic covariance, and the right plot showcases how tau estimation assists in dismantling horizontal pleiotropy from the vertical in the HVP model, even when all causal SNPs of trait **y** are in horizontal pleiotropy with trait **c**. Notably, the simulation employs a two-sample MR approach with sample sizes of 50,000 and 25,000 individuals, using 560 causal SNPs. The model specification is **y** = **c**⋅τ + **α** + **e**, where **c** = **β** + **ϵ**, and effects are drawn from a multivariate normal distribution, excluding τ. The variance-covariance structures for genetic and residual effects maintain **y**'s phenotypic variance at 1.


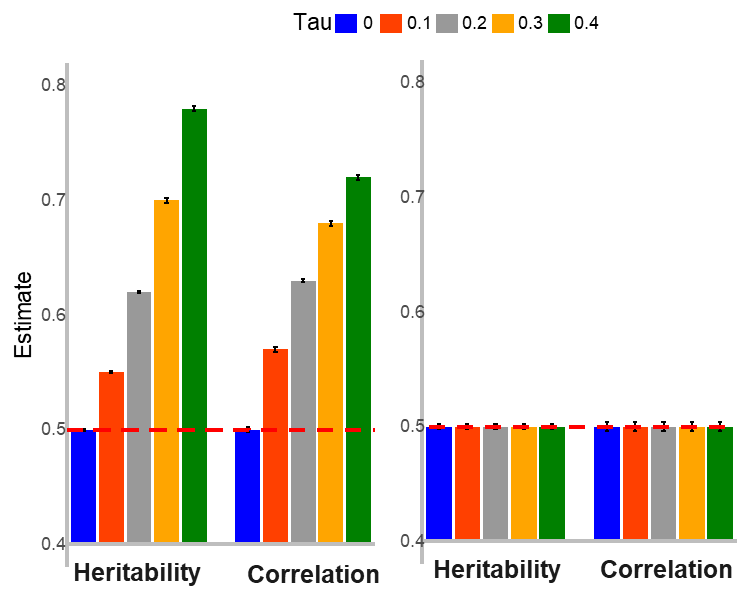


Supplementary Figure 2: Estimated Heritability(h^2^) and Genetic Correlation(r_g_). This figure presents results from a simulation examining the heritability of trait **y** and the genetic correlation between traits **y** and **c** in scenario 5 (Method). The left panel shows the biased estimates due to vertical pleiotropy. The right panel demonstrates that, after estimating τ, the HPV model successfully disentangle horizontal pleiotropy from the vertical pleiotropy, thereby correcting both heritability and genetic correlation estimates.


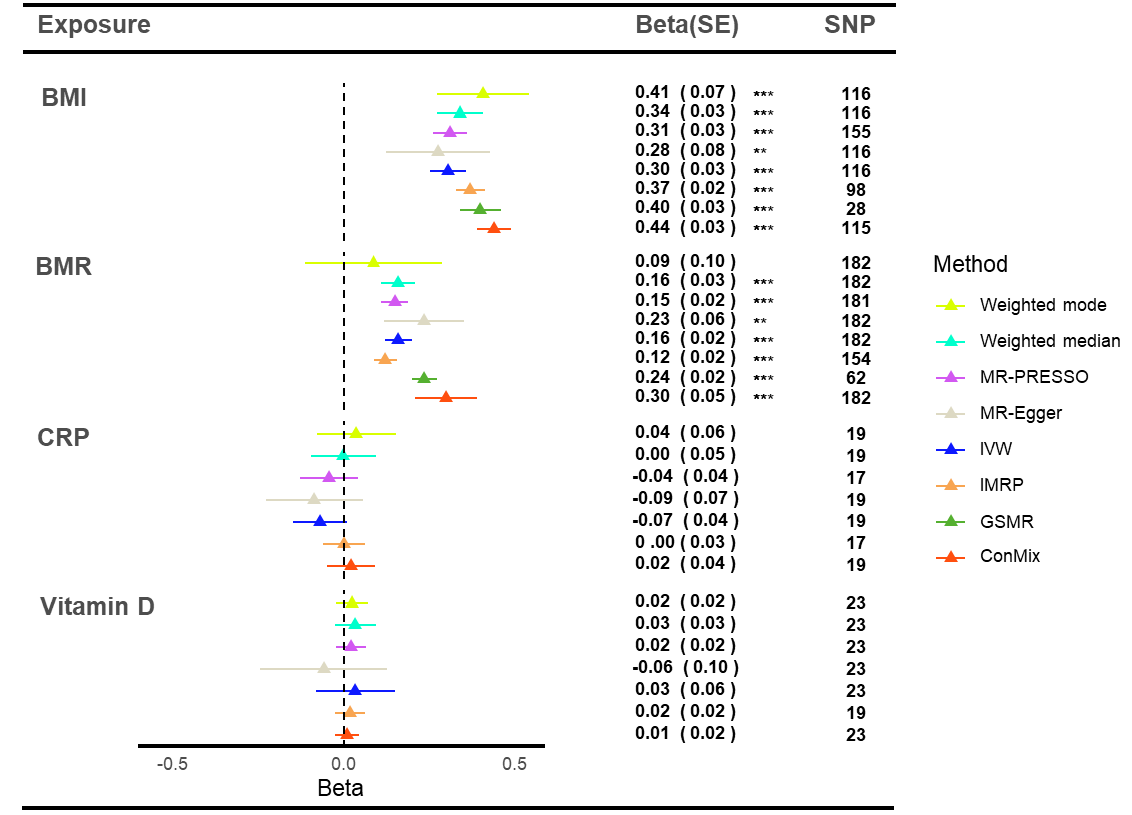


Supplementary Figure 3: Causal relationships between MetS as an outcome and BMR, BMI, CRP, and vitamin D as exposures. Abbreviations: MetS metabolic syndrome, BMI body mass index, BMR basal metabolic rate, CRP C-reactive protein, SE standard error, conMix contamination mixture, GSMR generalized summary data based Mendelian randomization, IMRP iterative Mendelian randomization and pleiotropy, IVW inverse variance weighted.

**
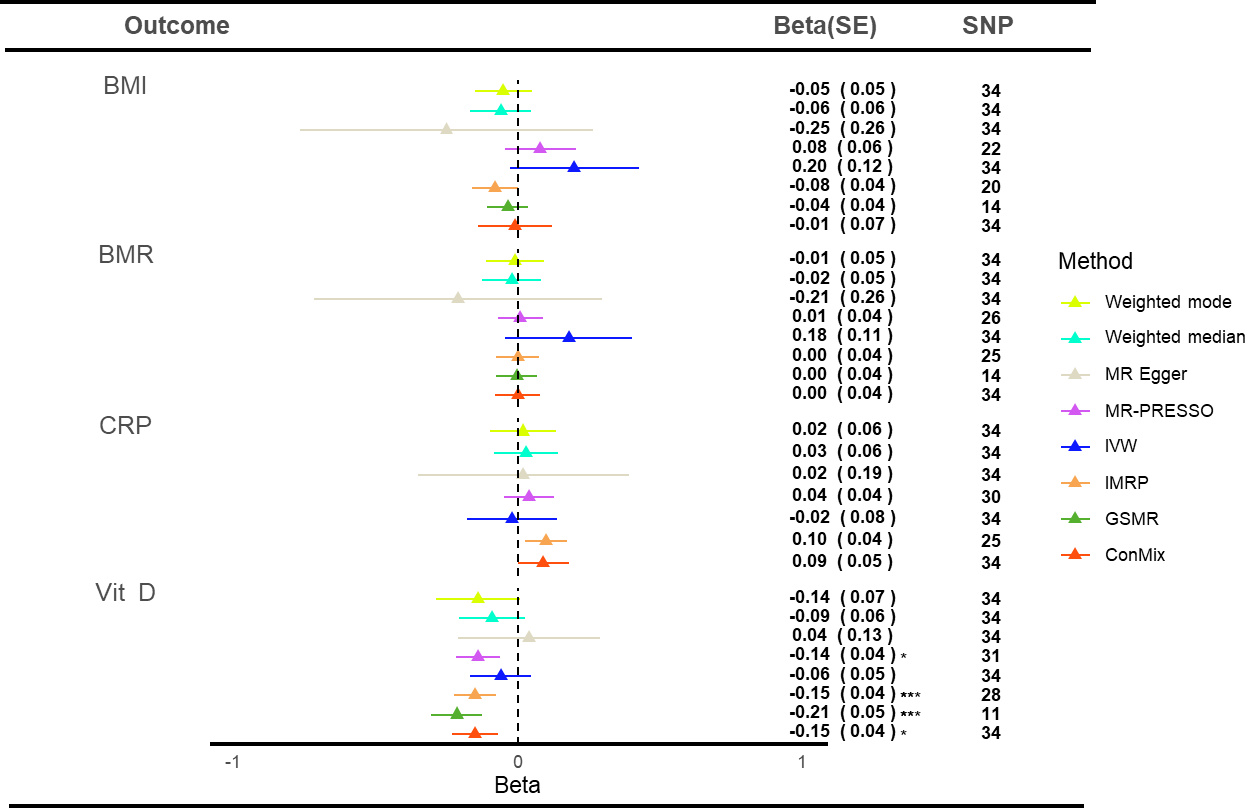
**

Supplementary Figure 4: Causal relationships between MetS as an exposure and BMR, BMI, CRP, and vitamin D as outcomes. Abbreviations: MetS metabolic syndrome, BMI body mass index, BMR basal metabolic rate, CRP C-reactive protein, SE standard error, conMix contamination mixture, GSMR generalized summary data based Mendelian randomization, IMRP iterative Mendelian randomization and pleiotropy, IVW inverse variance weighted.


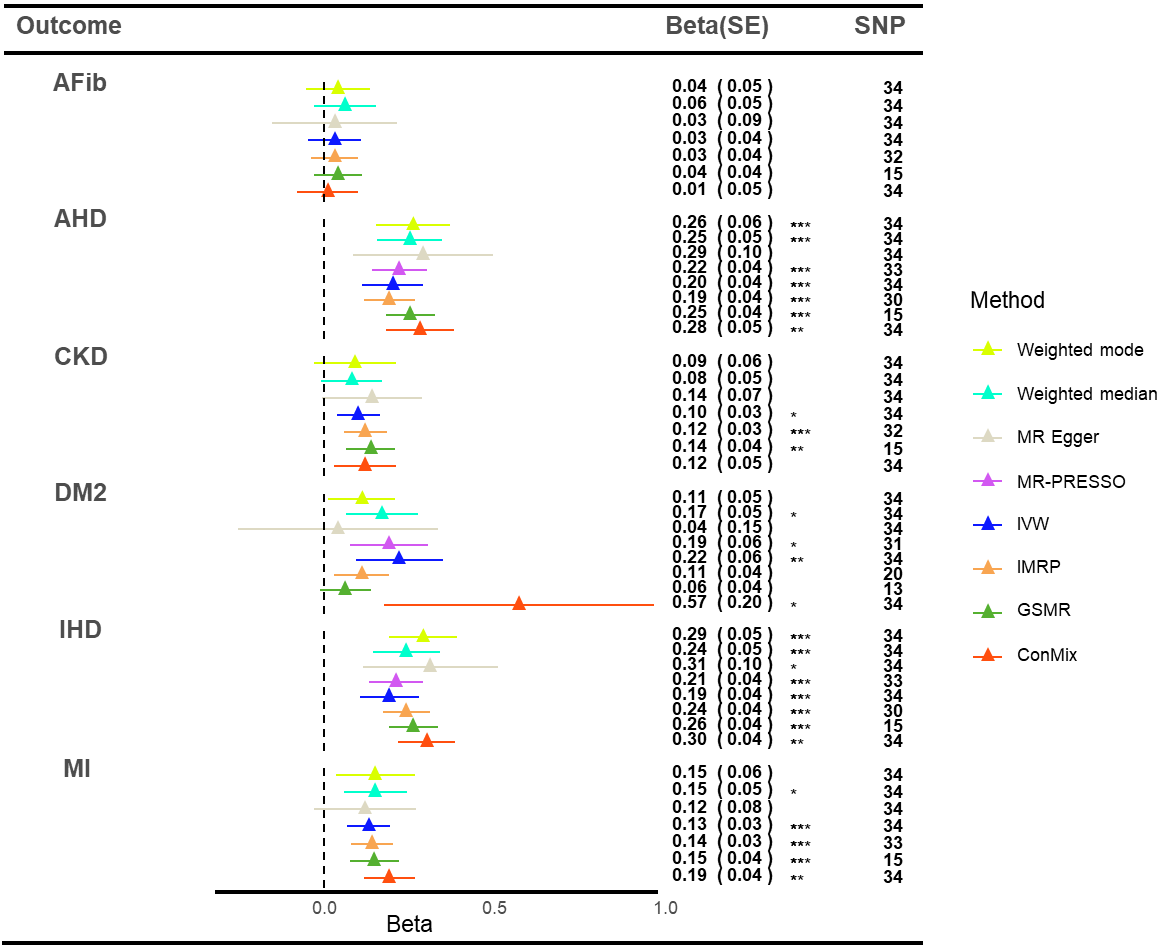


Supplementary Figure 5: Causal relationships between MetS as the exposure and ICD 10 comorbidities as an outcome. Abbreviations: MetS metabolic syndrome, AFib atrial fibrillation and or flutters, AHD atherosclerotic heart disease, IHD ischemic heart disease, MI myocardial infarction, CKD chronic kidney disease, and DM2 type II diabetes, SE standard error, conMix contamination mixture, GSMR generalized summary data based Mendelian randomization, IMRP iterative Mendelian randomization and pleiotropy, IVW inverse variance weighted.


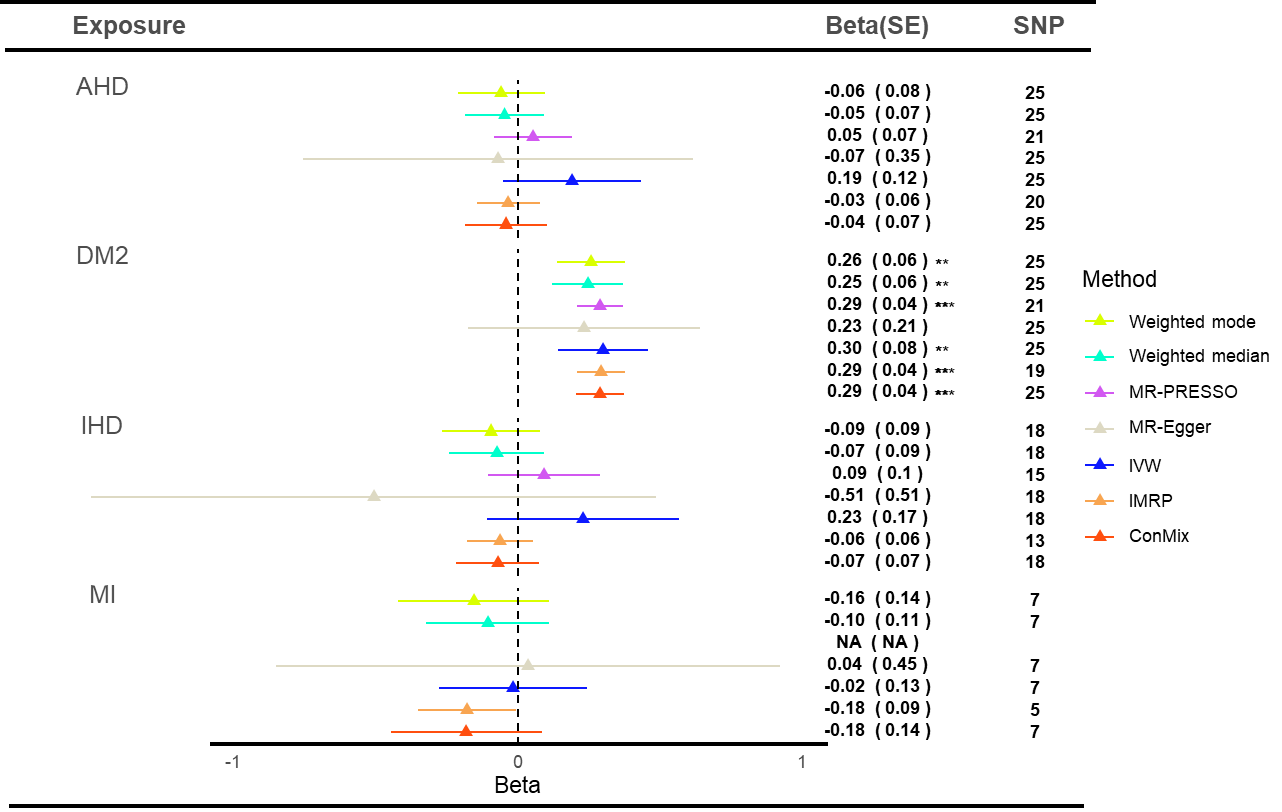


Figure 6: Causal relationships between MetS as an outcome and ICD 10 comorbidities as exposures. Abbreviations: MetS metabolic syndrome, AFib atrial fibrillation and or flutters, AHD atherosclerotic heart disease, IHD ischemic heart disease, MI myocardial infarction, CKD chronic kidney disease, and DM2 type II diabetes, SE standard error, conMix contamination mixture, GSMR generalized summary data based Mendelian randomization, IMRP iterative Mendelian randomization and pleiotropy, IVW inverse variance weighted.

1. Zhu, Z., Zheng, Z., Zhang, F., Wu, Y., Trzaskowski, M., Maier, R., Robinson, M.R., McGrath, J.J., Visscher, P.M., Wray, N.R., and Yang, J. (2018). Causal associations between risk factors and common diseases inferred from GWAS summary data. Nat Commun *9*, 224. 10.1038/s41467-017-02317-2.

2. Amente, L.D., Mills, N.T., Le, T.D., Hypponen, E., and Lee, S.H. (2025). A latent outcome variable approach for Mendelian randomization using the stochastic expectation maximization algorithm. Hum Genet *144*, 559-574. 10.1007/s00439-025-02739-9.
